# A diffusion model‐free framework with echo time dependence for free‐water elimination and brain tissue microstructure characterization

**DOI:** 10.1002/mrm.27181

**Published:** 2018-03-23

**Authors:** Miguel Molina‐Romero, Pedro A. Gómez, Jonathan I. Sperl, Michael Czisch, Philipp G. Sämann, Derek K. Jones, Marion I. Menzel, Bjoern H. Menze

**Affiliations:** ^1^ Department of Computer Science Technical University of Munich Garching Germany; ^2^ GE Global Research Europe Garching Germany; ^3^ Max Planck Institute of Psychiatry Munich Germany; ^4^ CUBRIC, Cardiff University Cardiff UK; ^5^ School of Psychology, Faculty of Health Sciences Australian Catholic University Melbourne Australia; ^6^ Institute for Advanced Study Technical University of Munich Garching Germany

**Keywords:** blind source separation, brain microstructure, diffusion MRI, free‐water elimination, MR relaxometry, nonnegative matrix factorization

## Abstract

**Purpose:**

The compartmental nature of brain tissue microstructure is typically studied by diffusion MRI, MR relaxometry or their correlation. Diffusion MRI relies on signal representations or biophysical models, while MR relaxometry and correlation studies are based on regularized inverse Laplace transforms (ILTs). Here we introduce a general framework for characterizing microstructure that does not depend on diffusion modeling and replaces ill‐posed ILTs with blind source separation (BSS). This framework yields proton density, relaxation times, volume fractions, and signal disentanglement, allowing for separation of the free‐water component.

**Theory and Methods:**

Diffusion experiments repeated for several different echo times, contain entangled diffusion and relaxation compartmental information. These can be disentangled by BSS using a physically constrained nonnegative matrix factorization.

**Results:**

Computer simulations, phantom studies, together with repeatability and reproducibility experiments demonstrated that BSS is capable of estimating proton density, compartmental volume fractions and transversal relaxations. In vivo results proved its potential to correct for free‐water contamination and to estimate tissue parameters.

**Conclusion:**

Formulation of the diffusion‐relaxation dependence as a BSS problem introduces a new framework for studying microstructure compartmentalization, and a novel tool for free‐water elimination.

## 1 INTRODUCTION

More than 50 years have passed since Stejskal and Tanner published their early research on pulsed gradient spin‐echo.^1^ Thereafter, diffusion weighted imaging became an essential tool for nondestructive tissue microstructure characterization. The pioneering studies on ex vivo tissue and simulations of Krägger,^2^ Latour et al.,^3^ Szafer et al.,^4^ and Stanisz et al.^5^ established the theoretical basis of the compartmental model of neural tissue.

These early contributions were later translated to target specific biomarkers for in vivo human studies. White matter (WM) anisotropy became fiber orientation with the introduction of diffusion tensor imaging (DTI).^6^ The composite hindered and restricted model of diffusion MR imaging (CHARMED)^7^ extended DTI to two compartments with restricted and hindered diffusion behavior. Using the same principles, the neurite orientation dispersion and density imaging (NODDI) model^8^ introduced fiber orientation dispersion metrics and added an isotropic compartment. Additionally, axon diameter was addressed by AxCaliber^9^ and ActiveAx.^10^ These and other approaches rely on diffusion signal representations or a variety of geometric biophysical assumptions about the underlying tissue compartments, producing a wide range of possible configurations.^11^

In parallel with the development of multicomponent diffusion tissue models, relaxometry addressed the compartmental nature of tissue microstructure from a different perspective.^12^ Multi‐echo spin echo (SE) experiments combined with regularized inverse Laplace transforms (ILTs) for multi‐exponential fitting showed the presence of multiple water compartments in the tissue. Nonnegative least squares (NNLS)^13^ is the current gold standard for computing a regularized discrete ILTs for several components.^14^, ^15^ Alternatively, the exponential analysis via system identification using Steiglitz‐McBride (EASI‐SM) for multicomponent estimation was introduced by Stoika et al.^16^, ^17^ Additionally, mcDESPOT,^18^ used a spoiled gradient‐recalled echo and a balanced steady‐state free precession to yield relaxation, volume fraction, and water exchange parameters for three compartments.

Nevertheless, the paths of diffusion MRI and MR relaxometry have become entangled over the years. Studies on ex vivo nerves with a diffusion‐weighted Carr‐Purcell‐Meiboom‐Gill (CPMG) sequence^19^, ^20^ showed the relationship that existed between compartmental *T*_2_ decay and diffusivity. However, diffusion‐weighted CPMG experiments need long acquisition times and high specific absorption rates, which makes them unsuitable for human in vivo studies. Typically, two‐dimensional ILTs were used to fit the data, but this approach is highly ill‐posed and requires large amounts of data for stabilization. Recently, Benjamini et al.^21^ introduced the marginal distributions constrained optimization (MADCO), a nonCPMG compressed‐sensing based solution that reduced the amount of data necessary for NMR diffusion–relaxation correlation experiments. Kim et al. translated diffusion–relaxation correlation spectroscopy (DR‐COSY)^22^, ^23^ into imaging (DR‐CSI)^24^ using spatial regularization to reduce the amount of necessary data and stabilize the ILTs. However, they require specific diffusion protocols with increasing *b*‐values along a unique diffusion direction and repeated echoes or inversion times. Other alternatives combine diffusion models with multicompartmental relaxation. For instance, inversion recovery diffusion weighted imaging has been used to identify fiber populations,^25^, ^26^ and WM integrity has been characterized using the axonal stick model and multiple echo times (TE).^27^

Compartmental analysis of the diffusion signal is intimately related to a recurring issue: cerebrospinal fluid (CSF) or free‐water contamination.^28^, ^29^ All the existing contributions agree on using a bi‐tensor signal model: parenchyma and CSF. However, this is an ill‐posed problem for a single‐shell and ill‐conditioned for multiple‐shell acquisitions.^30^ Spatial regularization was proposed by Pasternak et al.,^31^ relying on the local smoothness of the diffusion tensor. Later, a protocol optimization for multiple shells was presented by Hoy et al.,^32^ eliminating such a constraint. Other solutions regularize the problem by adding priors^33^ or finding the best fit to the model.^34^ Nevertheless, the CSF contribution to the diffusion signal depends on the TE. Thus, disentangling the tissue CSF volume fraction requires an approach that includes *T*_2_ compartmental dependencies.^33^, ^35^, ^36^

We propose a general framework for studying diffusion and relaxation characteristics in tissue microstructures. We call it general because it does not model the compartmental diffusion behavior. It replaces the ILTs by a blind source separation (BSS) technique, reducing the minimum number of distinct echo times required to the number of compartments in the tissue, less than for ILTs‐based methods. Other than the requirement to measure at more than one echo time, this framework is diffusion protocol‐agnostic, and can be used in combination with any protocol of interest. Our approach quantifies proton density, compartmental volume fractions, and transverse relaxation times. Importantly, it handles diffusion signals from each compartment independently, allowing for individual analyses, and thus performs CSF partial volume correction as a direct application.

## 2 THEORY

Following the Bloch–Torrey equation, we describe the diffusion signal as a weighted sum of the signals from the compartments comprising the tissue

(1) $$X(\text{TE},b,g)=S_{0}\sum_{i=1}^{M}f_{i}e^{-\frac{\text{TE}}{T_{2_{i}}}}S_{i}(b,g).$$

Where *b* summarizes the gradient effects^1^, ^37^ and **g** defines the gradient directions. Here, the compartmental diffusion sources $S_{i}(b,g)$ are weighted by their volume fraction, *f_i_*, TE, and $T_{2_{i}}$. The exponent (the ratio between TE and $T_{2_{i}}$) scales the contribution of each compartment to the acquired signal. Therefore, measuring at different TEs produces distinct diffusion signals^38^ with different weights from the compartmental signal sources.

As a result, the signal of a single voxel measured with a protocol that accounts for multiple echoes can be formulated as

(2) $$\left[\begin{array}{c} X_{1}({\text{TE}}_{1},b,g)\\ \vdots \\ X_{N}({\text{TE}}_{N},b,g) \end{array}\right]=S_{0}\left[\begin{array}{ccc} f_{1}e^{\frac{-{\text{TE}}_{1}}{T_{2_{1}}}} & \cdots & f_{M}e^{\frac{-{\text{TE}}_{1}}{T_{2_{M}}}}\\ \vdots & \ddots & \vdots \\ f_{1}e^{\frac{-{\text{TE}}_{N}}{T_{2_{1}}}} & \cdots & f_{M}e^{\frac{-{\text{TE}}_{N}}{T_{2_{M}}}} \end{array}\right]\left[\begin{array}{c} S_{1}(b,g)\\ \vdots \\ S_{M}(b,g) \end{array}\right],$$

where *X_j_* (*j* ∈ [1,N]) are the diffusion signals acquired for the *N* TEs. *f_i_* and $T_{2_{i}}$ (*i* ∈ [1,M]) are the volume fraction and *T*_2_ decay for the *i*th compartment, respectively, and *M* is the number of compartments.

Equation (2) can be expressed in matrix form as **X = AS**. This is a matrix factorization of the measurements, $X\in {\mathbb{R}}_{\geq 0}^{N\times n}$, into two new matrices: the mixing matrix, $A\in {\mathbb{R}}_{\geq 0}^{N\times M}$, which is defined by the experimental TEs, the compartmental volume fractions *f*, and *T*_2_ decays; and the sources matrix, $S\in {\mathbb{R}}_{\geq 0}^{M\times n}$, representing the diffusion sources in each sub‐voxel compartment. Interestingly, we noticed from the definition of **A** that the ratio between the experimental TEs and $T_{2_{i}}$ determines the direction (or slope for *N* = 2) of the *i*th column vector of the mixing matrix. Therefore:

(3) $$T_{2_{i}}=\frac{{\text{TE}}_{k}-{\text{TE}}_{l}}{\log\left(\frac{a_{li}}{a_{ki}}\right)},$$

where TE_*k*_ < TE_*l*_, and *a_ki_* and *a_li_* are the *k*th and *l*th elements of the *i*th column of the mixing matrix, respectively.

Additionally, diffusion is an attenuation contrast and as such, S(b=0)=1, allowing Equation (2) to be rewritten as

(4) $$\left[\begin{array}{c} X_{1}({\text{TE}}_{1},b=0,g)\\ \vdots \\ X_{N}({\text{TE}}_{N},b=0,g) \end{array}\right]=S_{0}\left[\begin{array}{ccc} e^{\frac{-{\text{TE}}_{1}}{T_{2_{1}}}} & \cdots & e^{\frac{-{\text{TE}}_{1}}{T_{2_{M}}}}\\ \vdots & \ddots & \vdots \\ e^{\frac{-{\text{TE}}_{N}}{T_{2_{1}}}} & \cdots & e^{\frac{-{\text{TE}}_{N}}{T_{2_{M}}}} \end{array}\right]\left[\begin{array}{c} f_{1}\\ \vdots \\ f_{M} \end{array}\right],$$

which, together with $\sum_{i=1}^{M}f_{i}=1$, allows us to solve for the volume fractions and proton density (*f_i_* and *S*_0_) when the number of measurements matches the number of compartments (*M* = *N*). Contrary, when there are more compartments than measurements (*M* > *N*), Equation (4) is undetermined and *f_i_* and *S*_0_ cannot be estimated.

**Figure 1:**
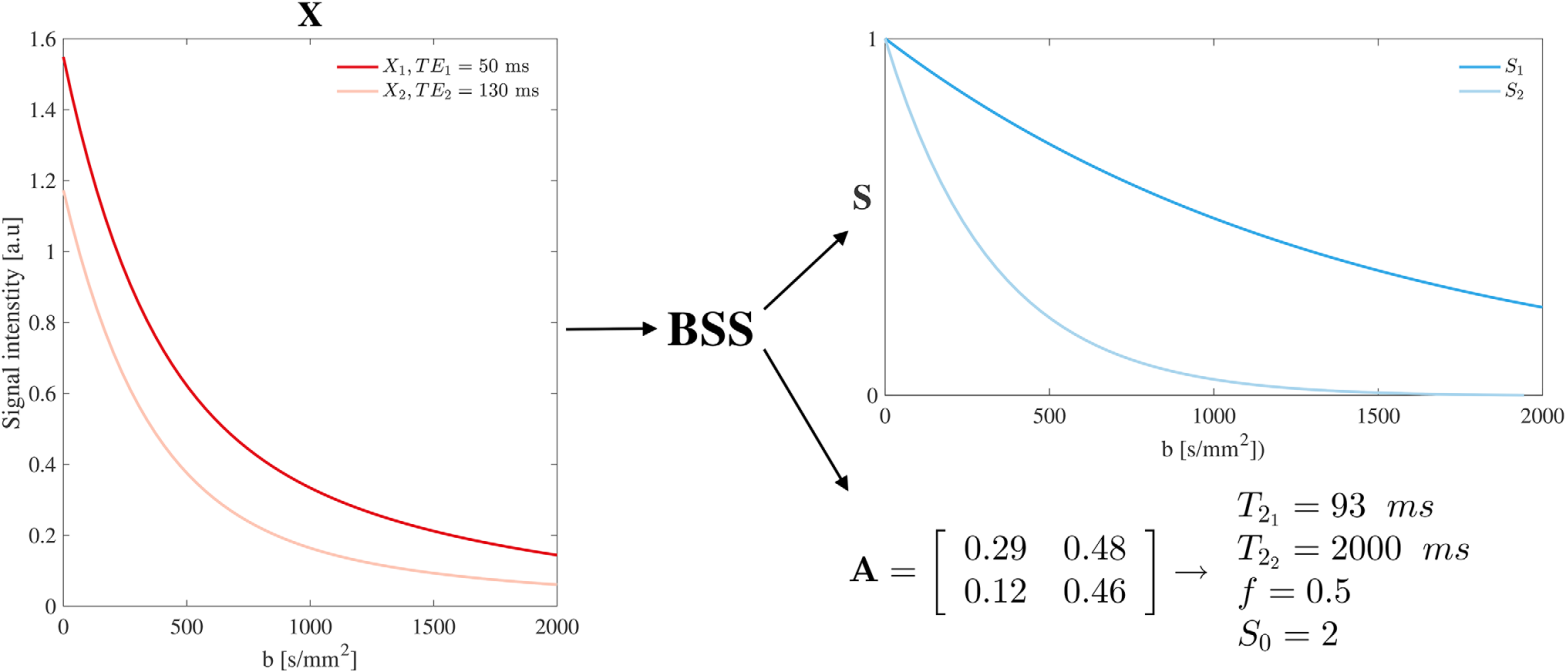
Factorization of measurements, **X**, into the sources, **S**, and mixing matrix, **A**. Example of a BSS operation for two mono‐exponential sources (*M* = 2) and two TE measurements (*N* = 2). In this illustration, the measurements, **X**, show a bi‐exponential decay profile. BSS is capable of separating these two independent exponential source functions, **S**; and calculating their mixing matrix, **A**. The parameters that determine the degree of mixing ( $T_{2_{1}},\;T_{2_{2}}$, and *f*), and the scaling factor, *S*_0_, were estimated as described in Equations (3) and (4). We showed an exponential case for simplicity, but BSS is not limited to this choice; any signal can be processed in the same manner

Factorizing **X** into **A** and **S** is known as BSS^39^ of mixed measurements into their generating sources (Figure 1). For BSS to identify these sources, they have to be distinct: $S_{i}\neq S_{j}$ ∀ i≠j. Therefore, based on previous work,^19^, ^20^ we assumed them to be different.

There are four main approaches to BSS: principal component analysis,^40^ independent component analysis,^41^ nonnegative matrix factorization (NMF),^42^ and sparse component analysis.^43^ Principal component analysis is not an applicable solution for this problem because the diffusion sources are not orthogonal. Independent component analysis assumes, as prior knowledge, that the signal sources are statistically independent and have nonGaussian distributions. However, diffusion MRI signals are correlated with the tissue structure and temperature and they present nonGaussian distributions only in restricted compartments, meaning that independent component analysis is not suitable either. We previously explored sparse component analysis^44^ and found that even though the results for simulations and real data for specific diffusion protocols were encouraging, finding a sparse and disjoint domain to meet the method's requirements was not always possible for arbitrary protocols. We observed the same issue for a version of NMF that enforces sparsity similarly.^36^

In the present work, we took a BSS approach based on NMF (assuming **X**, **A**, and **S** are nonnegative). Instead of depending on sparsity, we used a popular NMF solver: the alternating least squares algorithm (ALS).^42^, ^45^, ^46^ We chose ALS instead of the multiplicative update algorithm^47^ due to its faster convergence.^48^ We extended ALS to account for physically plausible limitations, resulting in Algorithm 1, which we refer to as constrained ALS (cALS). Compartmental *T*_2_ values available from the literature^15^ allowed us to limit the solution space of the columns of **A** (Equation (3)). Additionally, for in vivo data, the diffusion behavior of CSF is known to be approximately isotropic with $3\times 10^{-3}$ mm^2^/s diffusivity,^28^ adding extra prior information. These constraints and priors make cALS converge toward physically realistic solutions (Figure 1).

**Algorithm 1** Constrained Alternating Least Squares (cALS)

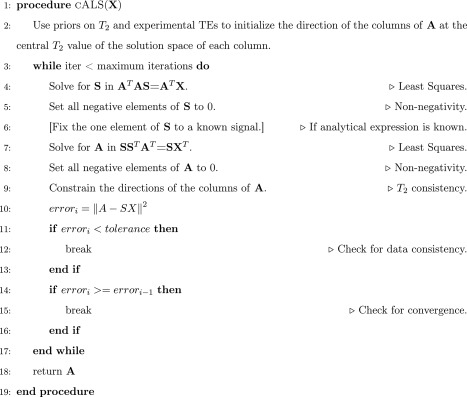

Constrained ALS initializes the column vectors of **A** at the central *T*_2_ of their given constraints, avoiding random initializations in regions that are not physically feasible and increasing the stability. After each iteration, cALS verifies that the resulting *T*_2_ of each column vector is between its boundaries, and sets it back to the center of its constrained solution space otherwise.

Following the factorization of **A**, we estimated *T*_2_ and *f* for each compartment, (Equations (3) and (4)), and recalculated the real **A**. This is important since the column norms of the factorized **A** do not tell us about the volume fractions. Then, **S = A^−1^X** is calculated.

An iterative algorithm like cALS inverts **A** repeatedly, requiring it to be nonsingular and introducing a new condition. From Equation (2), **A** is nonsingular when $T_{2_{i}}\neq T_{2_{j}}$ ∀ i≠j. Hence, in accordance with the literature,^19^, ^20^ we assumed that the transverse relaxation times for each compartment were distinct.

An open source implementation can be found in https://github.com/mmromero/dwybss.

## 3 METHODS

### 3.1 Simulations

NMF is known for converging to local minima.^45^ Thus, it is necessary to assess the impact of the constraints. We ran simulations with Rician noise for signal‐to‐noise ratio (SNR) levels of 50, 100, and 150 at the nondiffusion weighted volume and minimum TE. We accounted for *T*_2_ values, volume fractions, and diffusivities supported by literature.^15^, ^28^

### 3.2 Two compartments

Two compartments were simulated mimicking intra/extra‐axonal (IE) and CSF water. The diffusion protocol included one nondiffusion weighted volume and 30 directions. We modeled diffusion as a Gaussian process (see Supporting Information Figure S4). For all the simulations we used $T_{2_{\text{CSF}}}$ = 2000 ms, and varied $T_{2_{\text{IE}}}$ from 50 to 150 ms in 30 increments.^15^ Values of *f*_IE_ = 0.25, 0.5 and, 0.75 were used. We fixed TE_1_ = 60 ms, and explored TE_2_ from 70 to 150 ms in 31 increments. We defined ΔTE = TE_2_ − TE_1_. The performance of the cALS algorithm was tested under the following conditions:

1. **Overlapped *T*_2_ constraints**: $T_{2_{\text{IE}}}$ and $T_{2_{\text{CSF}}}$ were bounded from 0–1000 and 0–3000 ms, respectively, and no assumption on *S*_CSF_ was made (Figure 2 and Supporting Information Figure S5).
  **Figure 2:**
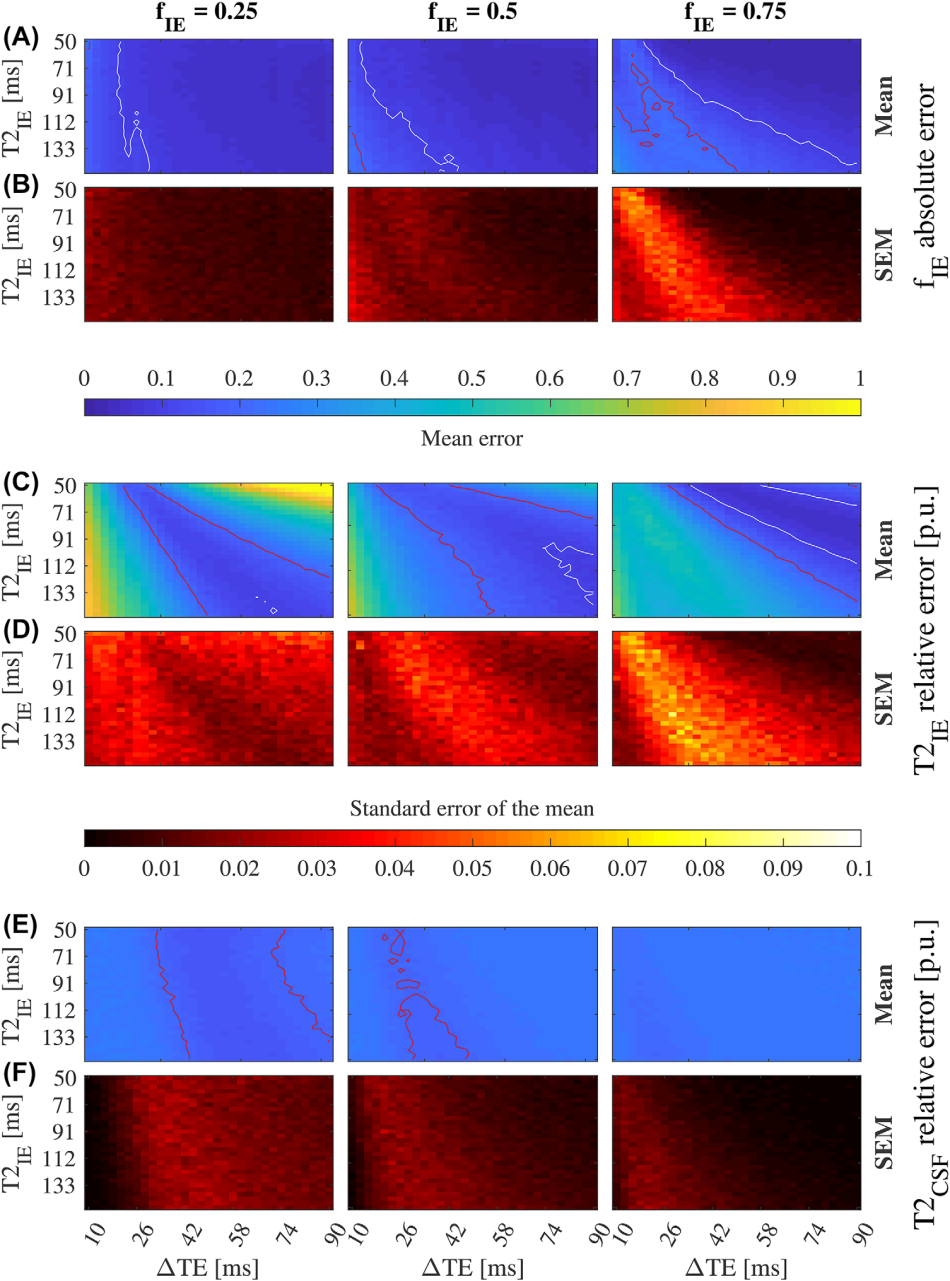
Convergence for two compartments (IE and CSF) with overlapping *T*_2_ constraints and no *S*_CSF_ prior (SNR = 50). The mean of *f*_IE_ absolute error and its standard error (SEM) (A, B), and the mean of $T_{2_{\text{IE}}}$ (C) and $T_{2_{\text{CSF}}}$ (E) relative errors per unit (p.u.), and their standard error (D, F). Red and white lines mark the 0.2 and 0.1 contour respectively. One thousand simulations were run for each combination of *f*_IE_, $T_{2_{\text{IE}}}$, and ΔTE. $T_{2_{\text{IE}}}$ and $T_{2_{\text{CSF}}}$ were bounded between 0–1000 ms and 0–3000 ms respectively, and no prior was imposed on *S*_CSF_. We defined the convergence area as the one with error lower than 0.1 for *f*_IE_ and $T_{2_{\text{IE}}}$. The bias of *f*_IE_ and $T_{2_{\text{IE}}}$ decreases for long ΔTEs as *f*_IE_ increases. See Supporting Information Figure S5 for more SNR levels
2. **Overlapped *T*_2_ constraints and prior *S*_CSF_**: $T_{2_{\text{IE}}}$ and $T_{2_{\text{CSF}}}$ were bounded from 0–1000 and 0–3000 ms, respectively. CSF diffusivity was assumed to be isotropic with value 3 × 10^−3^ mm^2^/s (Supporting Information Figure S10).
3. **Separated *T*_2_ constraints**: $T_{2_{\text{IE}}}$ and $T_{2_{\text{CSF}}}$ were bounded from 0–300 and 300–3000, ms, respectively, and no assumption on *S*_CSF_ was made (Supporting Information Figure S11).
4. **Separated *T*_2_ and prior *S_CSF_***: $T_{2_{\text{IE}}}$ and $T_{2_{\text{CSF}}}$ were bounded from 0–300 and 300–3000 ms, respectively. CSF diffusivity was assumed to be isotropic with value 3 × 10^−3^ mm^2^/s (Supporting Information Figure S13).
5. **Fixed** $T_{2_{\text{CSF}}}$: $T_{2_{\text{IE}}}$ was bounded from 0 to 300 ms. $T_{2_{\text{CSF}}}$ was fixed to 2000 ms. No assumption on *S*_CSF_ was made (Supporting Information Figure S12).
6. **Fixed** $T_{2_{\text{CSF}}}$ **and prior *S*_CSF_**: $T_{2_{\text{IE}}}$ was bounded from 0 to 300 ms. $T_{2_{\text{CSF}}}$ was fixed to 2000 ms. CSF diffusivity was assumed to be isotropic with value 3 × 10^−3^ mm^2^/s (Figure 3 and Supporting Information Figure S6).
  **Figure 3:**
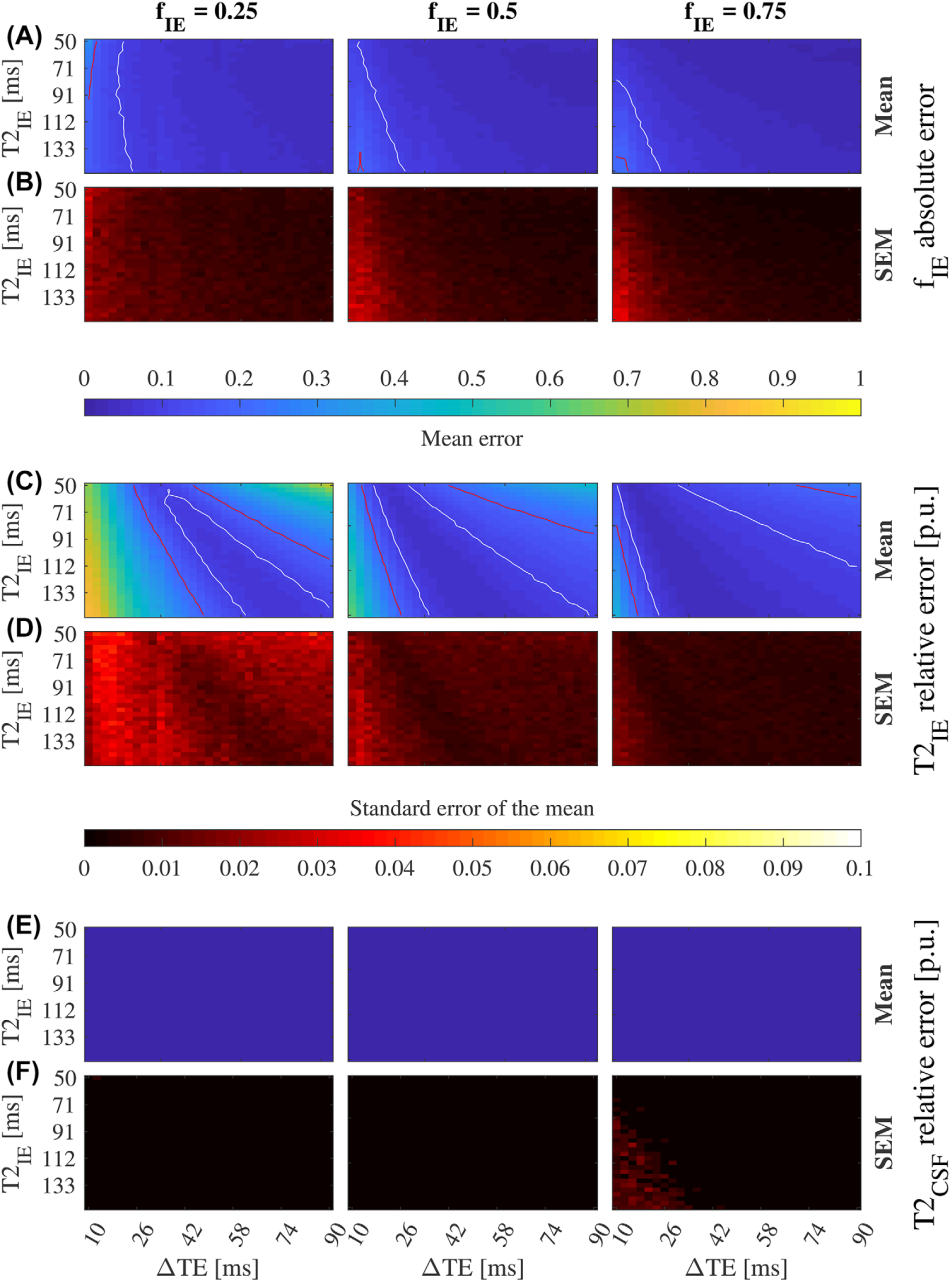
Convergence for two compartments (IE and CSF) with nonoverlapping *T*_2_ constraints and *S*_CSF_ prior (SNR = 50). The mean of *f*_IE_ absolute error and its standard error (SEM) (A, B), and the mean of $T_{2_{\text{IE}}}$ (C) and $T_{2_{\text{CSF}}}$ (E) relative error per unit (p.u.), and their standard errors (D, F). Red and white lines mark the 0.2 and 0.1 contour respectively. One thousand simulations were run for each combination of *f*_IE_, $T_{2_{\text{IE}}}$, and ΔTE. $T_{2_{\text{IE}}}$ and $T_{2_{\text{CSF}}}$ were bounded between 0–300 ms and 2000 ms, respectively, and *S*_CSF_ was set to have isotropic diffusivity with value 3 $\times 10^{-3}$ mm^2^/s. We defined the convergence area as the one with error lower than 0.1 for *f*_IE_ and $T_{2_{\text{IE}}}$. This area is larger than for Figure 2 stressing the importance of priors. See Supporting Information Figure S6 for more SNR levels

**Figure 4:**
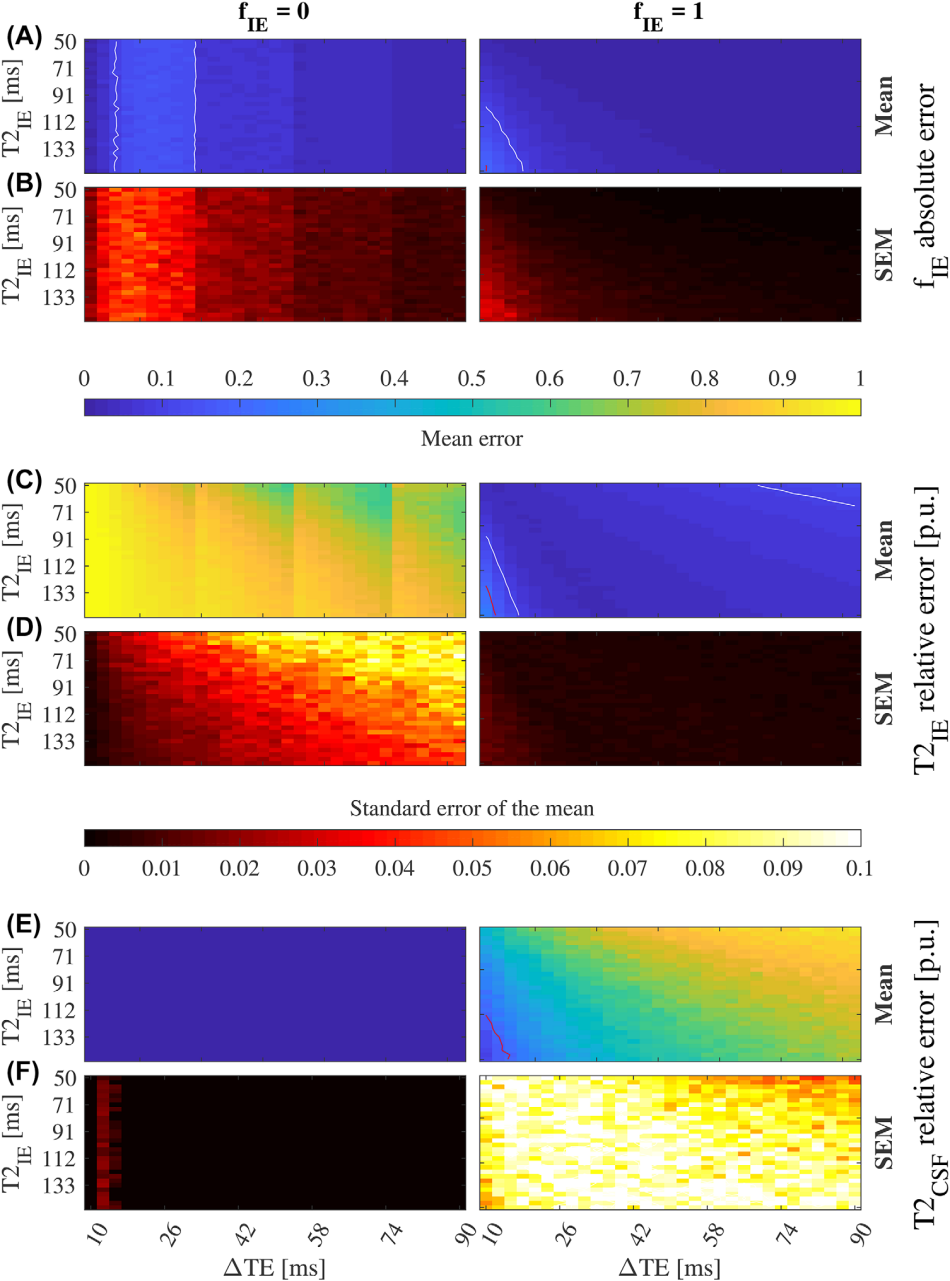
Convergence for two compartments (IE and CSF) with nonoverlapping *T*_2_ constraints and *S*_CSF_ prior when only one is actually present in the tissue (SNR = 50). The mean of *f*_IE_ absolute error and its standard error (SEM) (A, B), and the mean of $T_{2_{\text{IE}}}$ (C) and $T_{2_{\text{CSF}}}$ (E) relative error per unit (p.u.), and their standard errors (D, F). Red and white lines mark the 0.2 and 0.1 contour respectively. One thousand simulations were run for each combination of *f*_IE_, $T_{2_{\text{IE}}}$, and ΔTE. $T_{2_{\text{IE}}}$ and $T_{2_{\text{CSF}}}$ were bounded between 0–300 ms and 2000 ms, respectively, and *S*_CSF_ was set to have isotropic diffusivity with value 3 $\times 10^{-3}$ mm^2^/s. We defined the convergence area as the one with error lower than 0.1 for *f*_IE_ and $T_{2_{\text{IE}}}$. Estimates of *f*_IE_ are reliable for ΔTE > 45 ms (A, B). Estimates of $T_{2_{\text{IE}}}$ and $T_{2_{\text{CSF}}}$ are accurate for each case. See Supporting Information Figure S7 for more SNR levels

We repeated the last simulation for values of *f*_IE_ = 0 and 1, accounting only for IE or CSF (Figure 4 and Supporting Information Figure S7).

**Figure 5:**
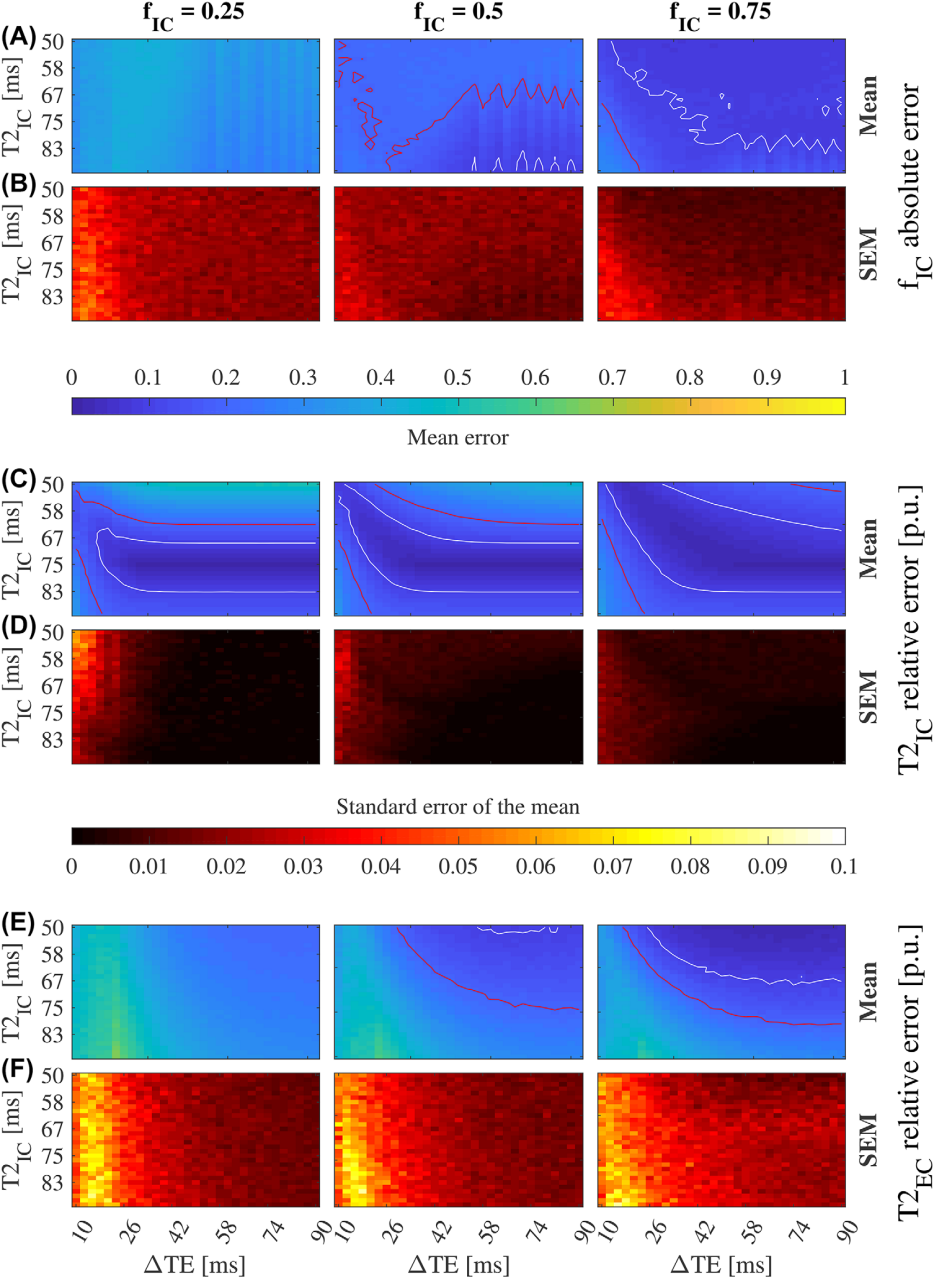
Convergence for two compartments (IC and EC) with overlapping *T*_2_ constraints and no other priors (SNR = 50). The mean of *f*_IE_ absolute error and its standard error (SEM) (A, B), and the mean of $T_{2_{\text{IE}}}$ (C) and $T_{2_{\text{CSF}}}$ (E) relative error per unit (p.u.), and their standard errors (D, F). Red and white lines mark the 0.2 and 0.1 contour respectively. One thousand simulations were run for each combination of *f*_IC_, $T_{2_{\text{IC}}}$, and ΔTE. $T_{2_{\text{IC}}}$ and $T_{2_{\text{EC}}}$ were bounded between 0–150 ms and 0–200 ms, respectively, and no other prior was imposed in the signal sources. We define the convergence area as the one with error lower than 0.1 for *f*_IC_, $T_{2_{\text{IC}}}$, and $T_{2_{\text{EC}}}$. Estimate of *f*_IC_ is biased for all *f*_IC_ levels. *T*_2_ estimates show a narrow band of convergence limited by the lack of prior knowledge (see Figure 2, Supporting Information Figures S5, S10) and the condition of **A** when the *T*_2_ values are similar. See Supporting Information Figure S8 for more SNR levels

Finally, intra‐cellular (IC) and extra‐cellular (EC) *T*_2_ values are similar.^15^ We assessed the potential of BSS to separate them. Two diffusion signals were generated (see Supporting Information Figure S14). We used *f*_IC_ = 0.25, 0.5, and 0.75. The $T_{2_{\text{IC}}}$ vales ranged from 50 to 90 ms in 30 increments, and $T_{2_{\text{EC}}}$ = 100 ms. TE_1_ was fixed to 60 ms and TE_2_ was varied between 70 and 150 ms in 31 increments. No assumption was made on the diffusion signals, and *T*_2_ constraints were defined between 0–150 and 0–200 ms for IC and EC, respectively (Figure 5 and Supporting Information Figure S8).

We simulated 1000 times each combination of parameters, and reported the mean value of the absolute error of *f*, the relative error of *T*_2_, and their standard errors (SEM).

### 3.3 Three compartments: Searching for myelin

We incorporated a fast decaying component to model myelin, and fixed the *T*_2_ of myelin ( $T_{2_{M}}$) to 15 ms.^15^ $T_{2_{\text{IE}}}$ was varied from 50 to 150 ms in 30 increments, and $T_{2_{\text{CSF}}}$ = 2000 ms. To account for short T2 components we needed to reduce the minimum TE of our simulations (see phantom experiments in the supporting information). Therefore, we fixed TE_1_ = 10 ms, TE_3_ = 150 ms, and varied TE_2_ from 20 to 140 ms in 31 increments. We defined ΔTE = TE_2_ − TE_1_. Three cases were explored: (1) *f*_M_ = 0.1, *f*_IE_ = 0.6; (2) *f*_M_ = 0.2, *f*_IE_ = 0.5; and (3) *f*_M_ = 0.3, *f*_IE_ = 0.4; keeping *f*_CSF_ = 0.3 for all of them. Simulations were run for two cases:

1. **Overlapped *T*_2_ constraints**: $T_{2_{M}},\;T_{2_{\text{IE}}}$, and $T_{2_{\text{CSF}}}$ were bounded from 0–40, 0–300, and 0–3000 ms, respectively. No assumption on *S*_CSF_ was made.
2. **Separated *T*_2_ constraints, fixed** $T_{2_{\text{CSF}}}$ **and prior *S_CSF_***: $T_{2_{M}}$ and $T_{2_{\text{IE}}}$ were bounded from 0–40 and 41–300 ms, respectively, while $T_{2_{\text{CSF}}}$ = 2000 ms. CSF diffusivity was assumed to be isotropic with value 3 × 10^−3^ mm^2^/s (Figure 6 and Supporting Information Figure S9).
  **Figure 6:**
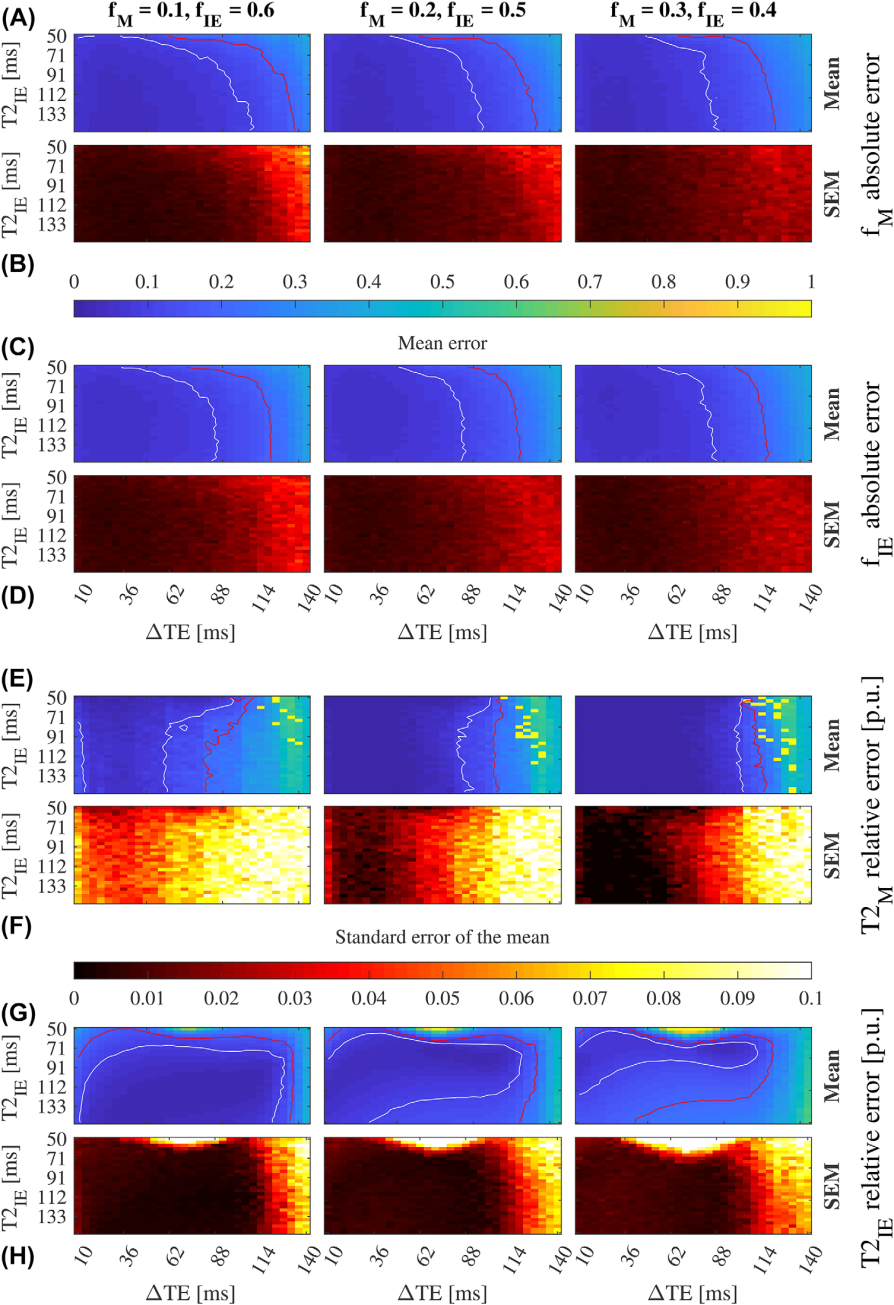
Convergence for three compartments (myelin, IE, and CSF) with nonoverlapping *T*_2_ constraints and *S*_CSF_ prior (SNR = 50). The mean absolute errors of the volume fraction estimates and their standard errors (SEM) (A–D); and the mean of $T_{2_{M}}$ (E) and $T_{2_{\text{IE}}}$ (G) relative error per unit (p.u.), and their standard errors (F, H). Red and white lines mark the 0.2 and 0.1 contour respectively. There is a large convergence area when TE_1_ = 10 ms, TE_2_ = 46 ms, and TE_3_ = 150 ms, which is not reachable with current clinical hardware. See Supporting Information Figure S9 for more SNR levels

Each combination of parameters was simulated 1000 times. The mean value of the absolute error of *f*, the relative error of *T*_2_, and their SEM were reported.

### 3.4 In vivo clinical data: Free‐water elimination

We aim to show that BSS has potential applications in clinical settings. To this end, we ran an experiment to analyze its performance for estimating tissue parameters and correcting for CSF contamination.

### 3.5 Data acquisition

Two volunteers, a male (age 28 years) and a female (age 24 years) were scanned in a 3.0 T GE MR750w (GE Healthcare, Milwaukee, WI). The in vivo study protocol was approved by our institutional review board and prior informed consent was obtained. We acquired seven diffusion pulsed gradient spin‐echo with echo planar imaging volumes for TE values from 75.1 to 135.1 ms in 10 ms increments. The following parameters were constant: FOV = 240 mm; 4 mm slice thickness; TR = 6000 ms; 96 × 96 matrix size; ASSET = 2; and 30 directions. Additionally, we measured fluid‐attenuated inversion recovery (FLAIR) SE echo planar imaging for 17 equally‐spaced TEs ranging from 20 to 260 ms. The same imaging parameters were used as for the diffusion experiments but with no acceleration (ASSET = 0).

### 3.6 Data analysis

**Figure 7:**
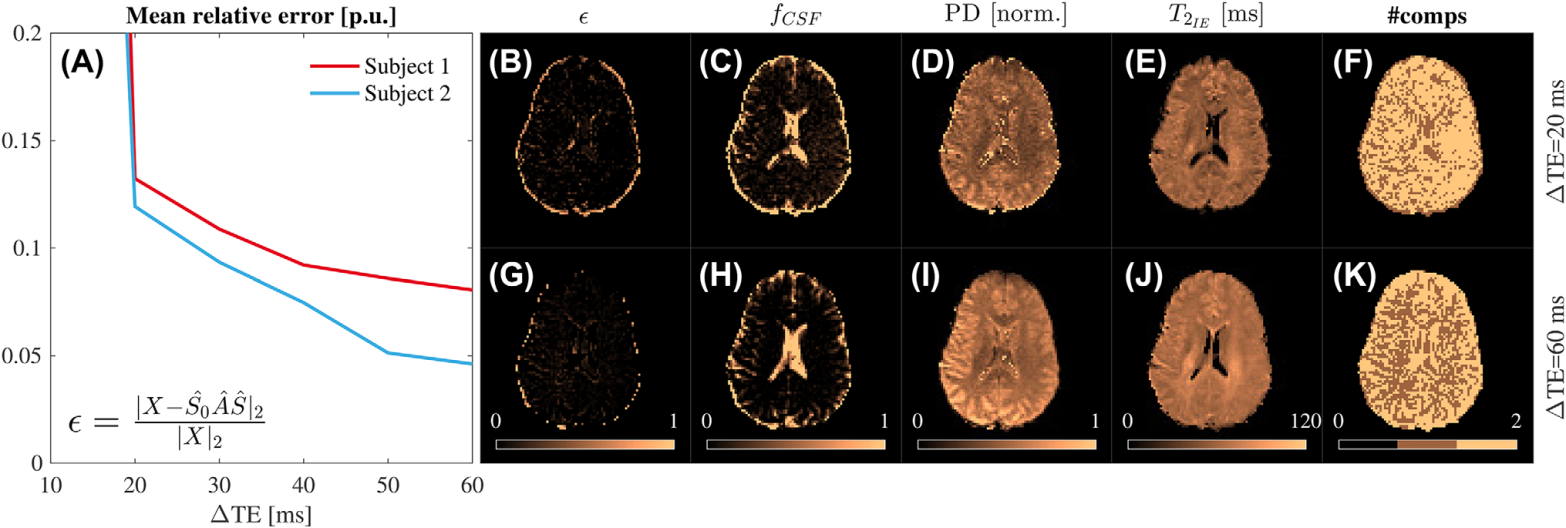
BSS relative factorization error for increasing ΔTE values. The evolution of the relative factorization error with ΔTE, averaged over the whole brain, is shown in (A). As an example of how this error reduction affects BSS estimates we also show the relative error maps (B) and (G), CSF volume fractions (C) and (H), proton densities (D) and (I), $T_{2_{\text{IE}}}$ values (E) and (J) and the number of compartments (F) and (K) for ΔTEs values of 20 and 60 ms. The mean relative factorization error decreases as ΔTE increases, improving the parameter estimates

**Figure 8:**
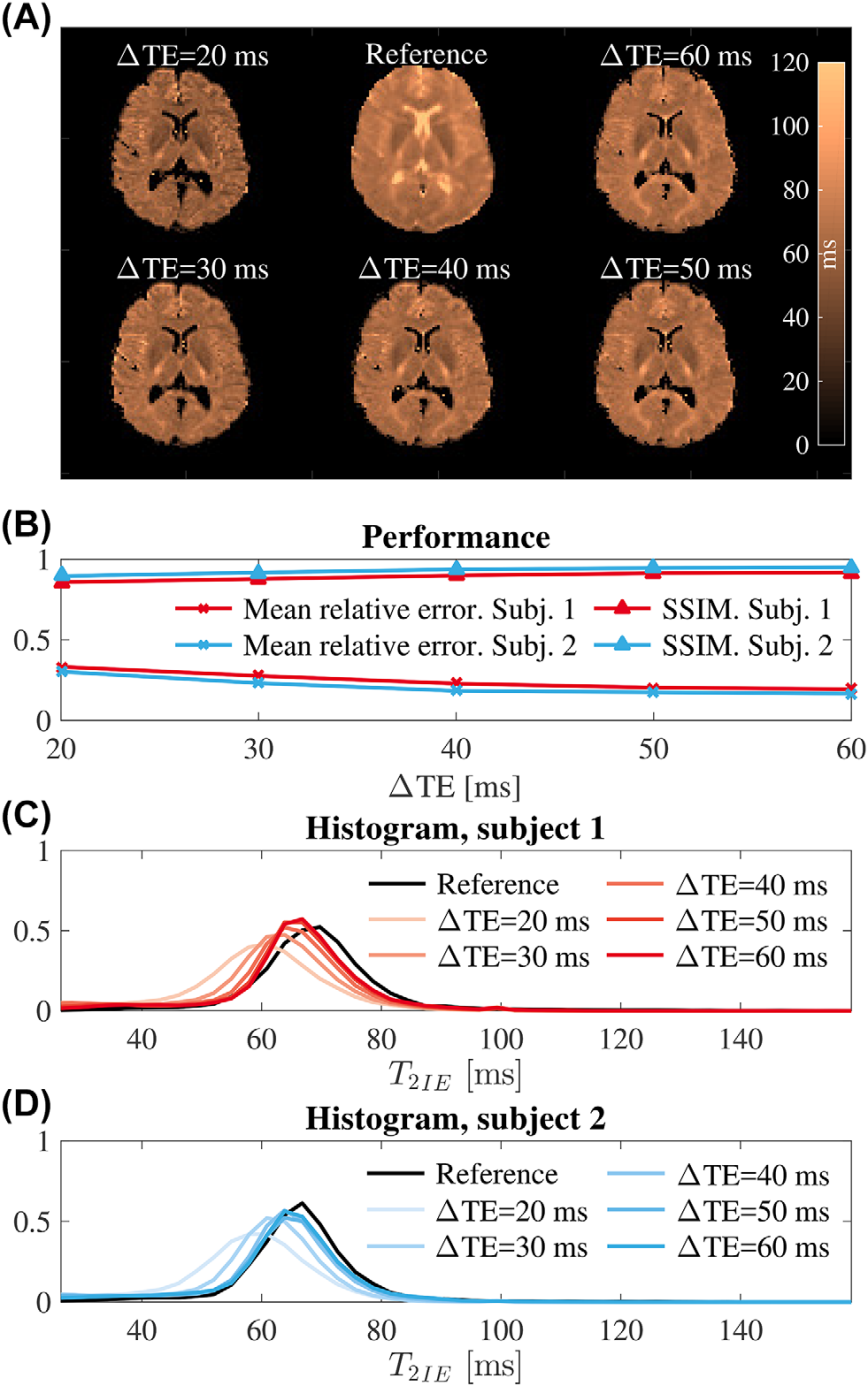
Comparison of the BSS‐estimated $T_{2_{\text{IE}}}$ values against a FLAIR reference. A comparison of the reference (A, upper middle), for subject one with the BSS $T_{2_{\text{IE}}}$ estimate is shown for increasing values of ΔTE. The visual comparison was quantified by SSIM^50^ and mean relative error (B). Histograms of the BSS‐estimated $T_{2_{\text{IE}}}$ values are plotted against the reference (C) and (D). High *T*_2_ values in the ventricles for the reference indicate that the suppression of the CSF signal in the FLAIR experiment was not perfect, although they appeared dark in the raw images. This might have induced a positive bias for the reference. Finally, the BSS‐estimated of $T_{2_{\text{IE}}}$ values for ΔTE above 50 ms showed good agreement with the reference

Diffusion data for all TEs were first registered with FSL FLIRT^49^ to the shortest TE volume. We then processed them with BSS in pairs ( M=N=2) with a fixed short TE of 75.1 ms. The long TE was increased from 85.1 to 135.1 ms for a total ΔTE of 60 ms (Figures 7 and 8). We used literature CSF values ( $T_{2_{\text{CSF}}}=2$ s and $D_{\text{CSF}}=3\times 10^{-3}$ mm^2^/s) as the prior knowledge, and constrained the possible values of $T_{2_{\text{IE}}}$ between 0 and 200 ms.^15^, ^28^ We report maps of the BSS relative factorization error (Figure 7A,B,G), CSF volume fraction (Figure 7C,H), proton density (Figure 7D,I), $T_{2_{\text{IE}}}$ (Figure 7E,J), and number of compartments (Figure 7F,K).

For reference, FLAIR multi‐echo echo planar imaging data were also registered with FLIRT to the shortest TE nondiffusion weighted volume. The signal decay for each voxel was then matched to a dictionary of mono‐exponential decays from 0 to 300 ms with a grid of 1 ms. We compared this map against the BSS $T_{2_{\text{IE}}}$ map (Figure 8).

We defined the relative error of the matrix factorization for the in vivo data as follows:

(5) $$\epsilon =\frac{|X-S_{0}AS{|}_{2}}{|X{|}_{2}}.$$

This is a measure of the performance of BSS for each voxel. Given that we calculated **S = A^−1^X**, this error formulation is sensitive to: (1) breaches of the BSS conditions due to artifacts, and (2) numerical instabilities due to the condition of **A**. Point one is the result of B_0_ drift, subject motion, flow, and eddy currents. These effects produce a violation of the BSS condition, making the signal sources different between TE measurements. The second point is the error amplification factor. A high ϵ denotes that the factorization could not find a solution within the constrained space and thus, results might not be trustworthy.

**Figure 9:**
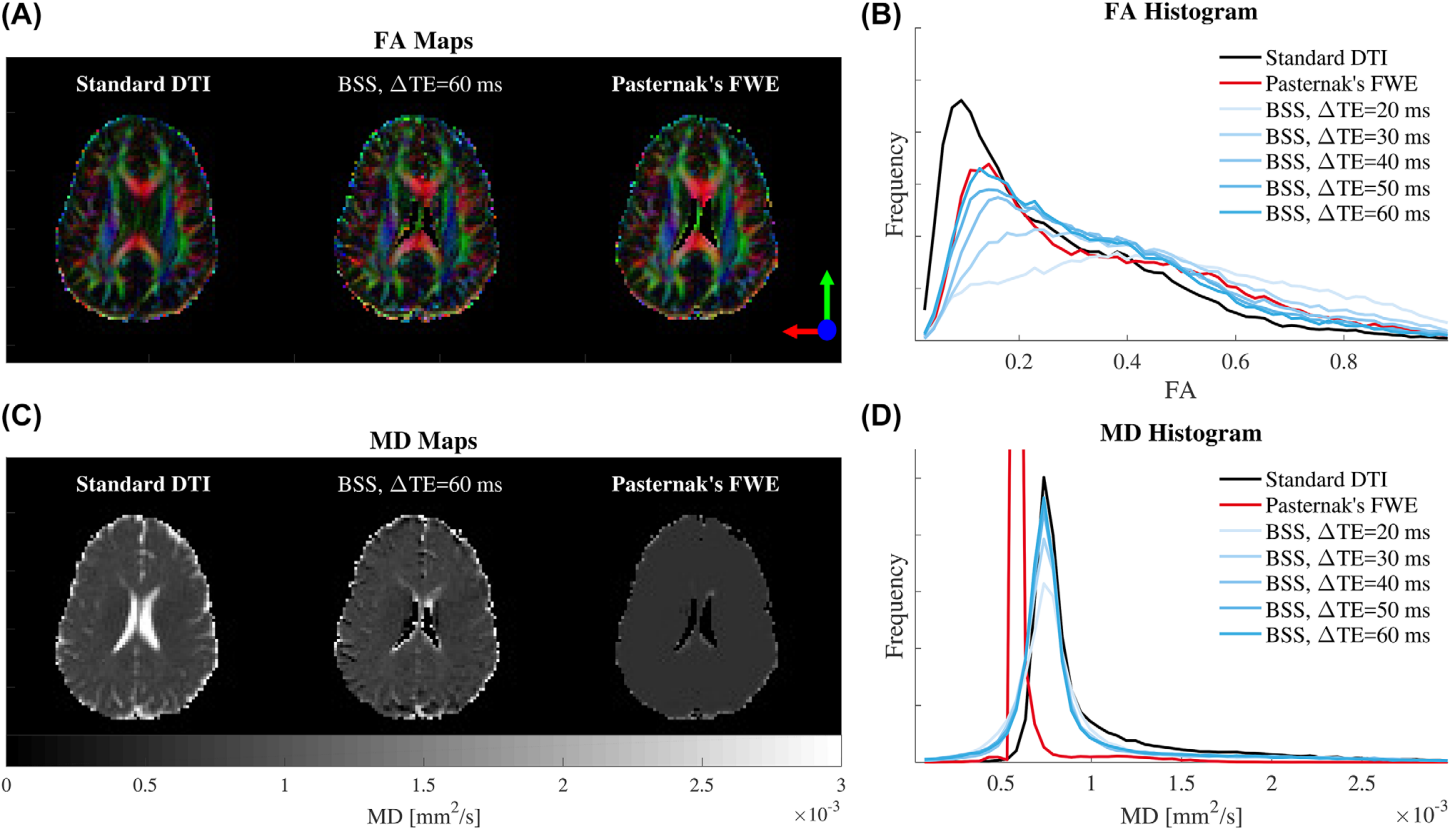
FA and MD of the BSS‐disentangled IE signal against the standard DTI and Pasternak's free‐water elimination (FWE) for subject two. Comparisons of the FA (B) and MD (D) histograms calculated from the separated IE signals are plotted against the standard DTI fit and Pasternak's method for the short TE measured data. MD (C) and colored FA (A) maps are also included for comparison. We observed a CSF correction effect in the long ΔTE BSS for FA in agreement with Pasternak's FWE. However, both method disagree for MD, where Pasternak's introduces spatial over‐regularization. See Supporting Information Figure S15 for the subject one

**Figure 10:**
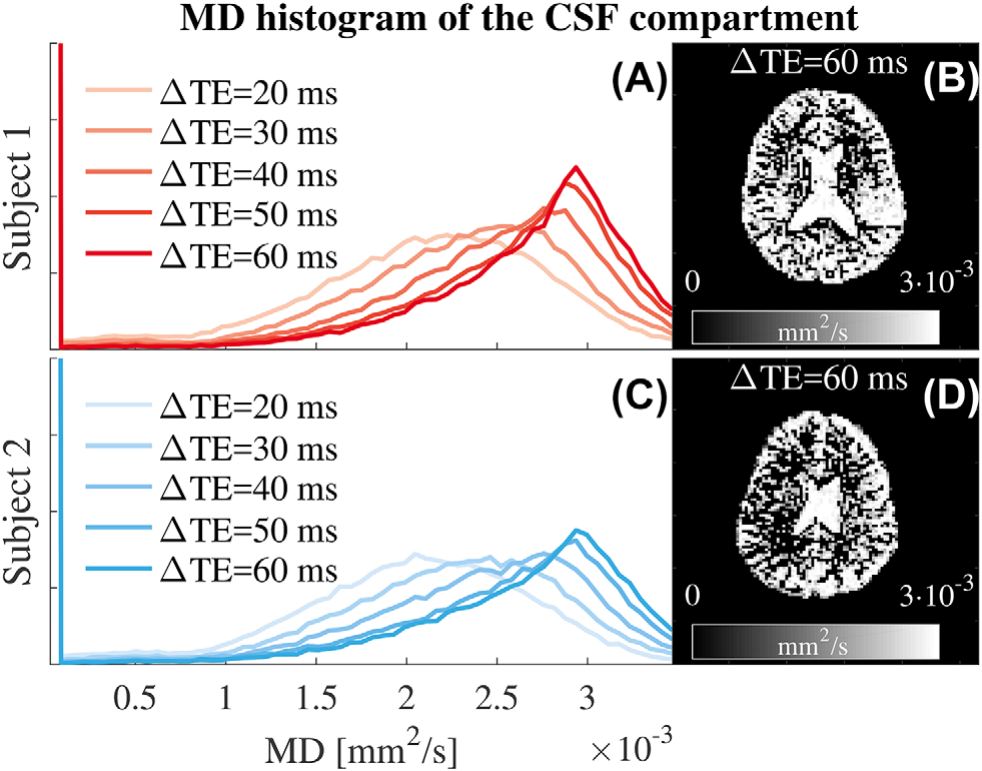
Evolution of the MD histogram of the BSS‐disentangled CSF component with ΔTE. The MD histograms, calculated from the DTI fits for the signals disentangled for the CSF compartment, are plotted in (A) and (C). MD maps (B) and (D) are shown for anatomical inspection. The CSF MD histograms tends toward 3 $\times 10^{-3}$ mm^2^/s, in agreement with the literature

Finally, BSS does not model the compartmental diffusion signal. However, to demonstrate a simple way to perform compartment‐independent analysis and correct for CSF contamination, we fitted the disentangled signals to the DTI model.^6^ We further fitted the measured diffusion volumes at the shortest TE, and the BSS separated signals for the IE and CSF compartments to a mono‐exponential model using standard linear regression (FSL FDT Toolbox (http://www.fmrib.ox.ac.uk/fsl)). For comparison, bi‐exponential models using Pasternak's and Collier's methods were used (Figures 9, 10, Supporting Information Figure S15). Fractional anisotropy (FA) and mean diffusivity (MD) maps were derived for each fit.

## 4 RESULTS

### 4.1 Simulations

#### 4.1.1 Two compartments

The convergence area is the region where the mean relative error of $T_{2_{\text{IE}}}$ is lower than 0.1 per unit (p.u). Its shape for all the simulations (Figures 2, 3, 4, 5, Supporting Information Figures S5, S6, S7, S8, S10, S11, S12, and S13) follows two effects. First, the condition number of the mixing matrix limits the lower bound of ΔTE: similar TE values produce more linearly dependent column vectors of **A**. And second, the SNR plays a double role, it increases the error regions where **A** is bad‐conditioned (small ΔTE), and limits the maximum ΔTE due to the *T*_2_ decay of the signals. Thus, when the SNR increases the convergence area grows and the region of minimum SEM, denoting an improvement on the stability of the algorithm. The convergence area also depends on the IE volume fraction. The larger is the contribution of IE, the better is the $T_{2_{\text{IE}}}$ estimate.

Adding priors on *S*_CSF_ improves the $T_{2_{\text{IE}}}$ estimate, even at SNR = 50 (Supporting Information Figure S10). Bounding the solution space into nonoverlapping regions also improves the results of $T_{2_{\text{IE}}}$ (Supporting Information Figure S11), although less than combining it with CSF prior knowledge (Supporting Information Figure S13). The $T_{2_{\text{CSF}}}$ estimate shows a 0.17 p.u. due to the small variation of *S*_CSF_ along the acquired TEs (4.4%). This is corrected when relaxometry prior is incorporated (Figure 3 and Supporting Information Figure S12). The comparison between Figures 2 and 3, show the benefit of including prior knowledge into the factorization algorithm, specially at low SNR. Then, the accuracy of the estimates will be influenced by the selection of ΔTE, the *T*_2_ boundaries, the *S*_CSF_ prior, and the expected $T_{2_{\text{IE}}}$ and *f*_IE_ values. We used literature values for $T_{2_{\text{IE}}},\;T_{2_{\text{CSF}}}$_,_ 15 and *S*_CSF._ 28 According to Figure 3A,B one needs a minimum ΔTE of 26 ms for an accurate *f*_IE_ estimate. Interestingly, *f*_IE_ is a reliable parameter that tell us about the bias of $T_{2_{\text{IE}}}$, the larger *f*_IE_ is, the more accurate $T_{2_{\text{IE}}}$ becomes (Figure 3A,C).

For one tissue compartment BSS is able to precisely (SEM < 0.01) estimate the volume fraction with mean absolute error below 0.1 when ΔTE > 35 ms (Figure 4A,B). When *f*_IE_ = 1 the area of mean convergence of the $T_{2_{\text{IE}}}$ estimate is almost independent from ΔTE (Figure 4C,D). We found an equivalent result for the mean relative error of $T_{2_{\text{CSF}}}$ when *f*_IE_ = 0 (Figure 4E,F), although in this case it comes from the $T_{2_{\text{CSF}}}$ prior. Notice the large error and instability of $T_{2_{\text{IE}}}$ and $T_{2_{\text{CSF}}}$ in the opposite cases, *f*_IE_ = 0 and *f*_IE_ = 1, respectively (Figure 4C,E). This results when BSS tries to find a component that is not in the tissue and thus, cannot be estimated.

For two components with similar *T*_2_ values and little priors (IC and EC) cALS losses efficiency. The volume fraction estimates are biased (Figure 5A), and $T_{2_{IC}}$ shows a narrow convergence region that is almost independent of ΔTE. The lower bound of this region is limited by the proximity of $T_{2_{\text{IC}}}$ and $T_{2_{\text{EC}}}$ that worses the condition of **A**. The upper bound results of the lack of prior on the signal of one of the compartments, in contrast with the *S*_CSF_ prior used before (compare Figure 2 and Supporting Information Figure S10) that increased the convergence area toward lower *T*_2_ values.

#### 4.1.2 Three compartments: Searching for myelin

The convergence area is the one where the errors of *f*_M_, *f*_IE_, $T_{2_{M}}$, and $T_{2_{\text{IE}}}$ are lower than 0.1 in absolute value for the volume fractions and per unit for *T*_2_. Figure 6A, C, E, G shows and optimal ΔTE = 36 ms. Notice that when ΔTE increases the error of the myelin parameters grows due to the reduction of the myelin contribution to the second TE, worsening the SNR of that component (Figure 6A,E). Since all the volume fractions add up to one, errors on *f*_M_ increase the error on *f*_IE_ (Figure 6A,C). The estimate of $T_{2_{\text{IE}}}$ is dependent on SNR and its volume fraction, compounding its calculation for SNR < 50 and *f*_IE_ < 0.4 (Supporting Information Figure S9G lower left corner).

One should notice that including a third compartment increases the condition number of **A**, rising the instability of the factorization (Figure 6F). See the phantom experiments in the Supporting Information.

### 4.2 In vivo clinical data: Free‐water elimination

We observed that the mean relative error for the whole brain ( 〈ϵ〉) decreased as ΔTE increased (Figure 7A,B,G), in agreement with phantom findings (see supporting information) and the results of the simulations for two compartments. Interestingly, for the maximum ΔTE, we can see that the number of compartments is two in regions next to the ventricles and the cortex, but one inside the ventricles and in some deep WM areas (Figure 7K). It is also noteworthy that the pure CSF areas (eg, the ventricles) have been removed from the $T_{2_{\text{IE}}}$ map (Figure 7E,J), while the opposite is observed in the CSF volume fraction (Figure 7C,H), indicating a successful disentangling effect.

We compared the BSS‐estimated $T_{2_{\text{IE}}}$ maps for increasing ΔTE values with the reference map obtained from the FLAIR multi‐echo SE data. We noted how the structural similarity index^50^ increased and the mean relative error decreased as ΔTE grew (Figure 8A,B). Additionally, the histograms for both subjects tended toward the reference as the difference between the short and long TEs grew. This reflects an underestimation of $T_{2_{\text{IE}}}$ for small ΔTE values that can be explained by Equation (3) and Supporting Information Figure S1C. Moreover, the FLAIR *T*_2_ map showed high values in the ventricles, possibly indicating imperfect CSF suppression and, thus, slightly increased reference values (Figure 8A,C,D).

FA and MD maps and histograms were calculated from the BSS IE and CSF disentangled signals for both subjects (Figures 9, 10, Supporting Information Figure S15). These maps displayed an overestimation of the CSF volume fraction for low ΔTE values (the low FA peak in Figure 9B and Supporting Information Figure S15B was removed). This resulted in a compensation effect for the previously shown underestimation of $T_{2_{\text{IE}}}$. Additionally, the FA histograms (Figure 9B and Supporting Information Figure S15B) showed a tendency toward higher FA values and a reduction of the low FA peak associated with free‐water. At long ΔTE values, FA seems to tend toward a stable distribution. We also observed an enlargement of the corpus callosum and a general recovery of peripheral WM tracts and the fornix in the colored FA maps (Figure 9A and Supporting Information Figure S15A).

Additionally, on the MD histograms for IE water (Figure 9D and Supporting Information Figure S15D) we found a reduced number of voxels with diffusivities greater than $1\times 10^{-3}$ mm^2^/s. In contrast, the main peak at 0.7 $\times 10^{-3}$ mm^2^/s, associated with the parenchyma, remained in its original position, indicating that IE water represents a nonCSF tissue. This MD reduction was also visible in the maps (Figure 9C and Supporting Information Figure S15C). Finally, the MD histograms for CSF water (Figure 10) showed a tendency toward $3\times 10^{-3}$ mm^2^/s as ΔTE increased, in agreement with the literature.^28^ All these findings agreed with a disentangling of IE and CSF signals and thus, a correction of the free‐water partial volume effect in the diffusion signal.

## 5 DISCUSSION

### 5.1 Stability

Four main approaches exist for the BSS problem (independent component analysis, principal component analysis, NMF, and sparse component analysis). Choosing the appropriate method depends on the prior knowledge of the signal sources. In our experiments, we relied on NMF, using a constrained version of the ALS algorithm (cALS). Others explored these algorithms before. Pauca et al.^51^ used low‐rank and sparsity constraints to distinguish semantic features in text mining, and later^52^ smoothness regularization to identify space objects from spectral data. Gao and Church^53^ also employed sparseness for cancer class discovery through gene clustering, which was later extended by Kim and Park^54^ improving the balance between accuracy and sparseness through regularization. They also introduced a variation based on the active set method^55^ and low‐rank approximation.^56^ Liu et al.^57^ incorporated label information to create a semi‐supervised matrix decomposition method. Sun and Févotte^58^ introduced a version based on the alternating direction method of multipliers^59^ (ADMM), that was further stabilized by Zhang et al.^60^

Supported by previous work, we presented a biophysical inspired solution to constrain the diffusion‐relaxometry NMF compartmental problem. Essentially, our cALS algorithm imposes two constraints: (1) the rows of **A** must follow exponential relationships (relaxometry); and (2) when the analytical expression of one component is known (ie, CSF) the corresponding row in **S** is fixed (diffusion). The stability of cALS is linked to the condition of **A** and SNR; an ill‐conditioned mixing matrix will lead to error propagation due to numerical instability. We optimized the experimental TEs to reduce the condition number of **A** for literature values of *T*_2_. However, further research based on ADMM might yield better results.

We ran extensive simulations for two compartments at clinical TE values with different priors, and three compartments at lower TEs. These simulations highlighted the importance of choosing literature supported priors to improve the convergence, especially at low SNR. Constrained ALS converges when the number of compartments in tissue is equal or lower than the expected, but it looses performance for species with similar *T*_2_.

Phantom experiments (see supporting information) agreed with simulation results, validating that BSS was able to accurately estimate *T*_2_ for one compartment and separate diffusion signal sources and estimate *T*_2_ and *f* for two compartments. However, they also showed that scaling the cALS algorithm to three compartments, including fast *T*_2_ decaying species, is unstable in the range of the clinically available TE values.

Finally, repeatability and reproducibility analyses (see supporting informtaion) show that cALS yield consistent results across repetitions and subjects, highlighting its stability.

### 5.2 Relaxation time and volume fraction estimates

BSS provides the means to estimate *T*_2_ relaxation values and volume fractions. Interestingly, only a number of TE repetitions equal to the number of compartments that are assumed to be in the tissue is necessary. This results of the substitution of the ILTs by BSS, in comparison to other techniques.^15^, ^17^, ^21^, ^24^ We found a good agreement between the $T_{2_{\text{IE}}}$ estimates of the FLAIR multi‐echo SE for 17 TEs and those of BSS for 2 TEs. In this sense, all the measurements along the diffusion space are considered for both TEs, incorporating redundancy and reinforcing the estimation of *T*_2_. The SNR for the in vivo data were 147 and 104 for subjects one and two. According to the simulations at ΔTE = 60 ms, the expected absolute error for the volume fraction estimate is below 0.03, meaning that $T_{2_{\text{IE}}}$ is highly reliable in WM areas, and lesser in the CSF borders.

### 5.3 Myelin detection

Simulations proved that our method has the potential to disentangle three compartments by reducing the minimum TE in diffusion experiments. As a result, myelin water could be incorporated into the model (Figure 6). However, we are prevented from conducting such experiments by gradient performance on clinical scanners.

### 5.4 Disentangling the diffusion sources and free water elimination

Unlike other multicompartment diffusion models^2^, ^7^, ^8^, ^11^ or more recent contributions,^27^, ^35^ our approach does not model compartmental diffusion. Our framework instead relies on three assumptions: (1) microstructural water compartments have distinct *T*_2_ relaxation times;^14^, ^15^ (2) each have different diffusion characteristics;^19^, ^20^ and (3) the effects of the water exchange are negligible on the timescale of our experiments.^9^, ^61^ Furthermore, our solution is diffusion protocol‐agnostic (only two TEs and one nondiffusion weighted volume are necessary), allowing for flexibility in the design of the acquisition protocol, which might include any number of diffusion directions and *b*‐values. This gives it an advantage over diffusion–relaxation correlation techniques based on regularized ILTs.^21^, ^24^

A promising application of the protocol‐agnostics nature of our framework is correcting for free water contamination. Recently Collier et al.^35^ included TE dependence in their bi‐exponential diffusion tensor model to regularize the fitting problem. However, they fitted the bi‐exponential DTI model directly. Contrary, our solution does not assume any particular diffusion model, we instead separated the signal from each compartment, allowing more flexible and independent study. In this regard, analysis of the signal associated with the CSF compartment can be seen as a disentanglement quality assurance metric (Figures 9, 10, Supporting Information Figure S15), or in brain tissue applications, a general indicator of the goodness‐of‐fit for IE and CSF.

We fitted our data to Collier's model^35^ without reaching convergence, which resulted due to our single‐shelled dataset. Comparison of BSS with Pasternak's free‐water elimination method^31^ is show in Figure 9 and Supporting Information Figure S15. We observed a good agreement between BSS for ΔTE = 60 ms and Pasternak's free‐water elimination for FAs between 0‐0.2 and 0.8‐1. In the middle FA range both methods disagree, BSS shows an homogeneous correction, while Pasternak's results follow the standard DTI fit from 0.2 to 0.4 and shows a correcting effect from 0.4 to 1 (Figure 9A,B, Supporting Information Figures S15A and S15B). It is impossible to determine which method is better (no ground‐truth). However, there are two indicators that BSS might be performing better: (1) the BSS FA curve runs in parallel to the standard DTI fit from 0.2 to 0.8, denoting an stable correction without favoring any FA range; and (2) Pasternak's MD is spatially over‐regularized (Figure 9C,D, Supporting Information Figures S15C and S15D), while BSS's MD keeps its maximum at 0.7 mm^2^/s, the reference for parenchyma.^28^

Long ΔTE values benefit our framework, which is not surprising and agrees with the findings of Collier et al.^35^ This is not only due to the relationship between **A** and *T*_2_ (Equation (3) and Supporting Information Figure S1C) but also because longer differences between TEs produce more distinct levels of mixing and thus better codification of the information from each source. That is to say, the short TE contains more information about the fast‐relaxing species, while the long TE is dominated by CSF.

## 6 CONCLUSIONS

We have introduced for the first time a BSS framework for expressing the relationships between diffusion signals acquired at different TEs. This new approach does not rely on diffusion modeling or the ILT. Our results show that, with the current hardware, blind source separation allows for disentangling the diffusion signal sources generated by each sub‐voxel compartment up to two compartments, making it a suitable tool for free‐water elimination. Moreover, it simultaneously estimates proton density, volume fractions, relaxation times and the number of compartments in the underlying microstructure, paving the way for tissue microstructure characterization when the hardware constraints are relieved.

## CONFLICT OF INTEREST

Miguel Molina Romero and Pedro A. Gómez receive research support by GE Global Research. Dr. Jonathan I. Sperl was GE Global Research employee during the process of this work and currently is Siemens Healthcare employee. Dr. Marion I. Menzel is GE Healthcare employee.

## Supporting information

Additional Supporting Information may be found online in the supporting information tab for this article.

**FIGURE S1** Evolution of the relative error in the *T*_2_ estimate with ΔTE for one compartment. The mean relative error of *T*_2_ estimated using BSS is shown in (a) for NNLS and in (b) for EASI‐SM references. ΔTE goes from 5 ms (darker colors) to 50 ms (lighter colors). The dependence of *T*_2_ on the direction (slope) of the columns of **A** (Equation 3) is shown in (c), where it can be seen how increasing ΔTE improves the dynamic range of the slope of **A**, resulting in a better estimate for *T*_2_. Except for ROI_1_ and ROI_11_, the remaining ones reduce the *T*_2_ mean relative error as ΔTE increases (a and b, lighter colors are closer to zero), in agreement with plot c.
**FIGURE S2** Separation of two compartments and parameter estimation for the phantom data. The signal sources of the *simulated* dataset are plotted in (a), and the *measured* data generated from the sources in (b). The resulting mixtures for both datasets are shown in (c). We use the subscripts *M* and *S* to refer to estimates for the *measured* and *simulated* datasets, respectively. Measurement errors are highlighted by the differences between the *measured* and *simulated* signals, shown in (c). BSS disentangled the original sources for both datasets, as shown in (d). We chose a ΔTE of 50 ms to minimize the condition of **A** (shown in (e)) and increase the numerical stability of the framework. Finally, the relative errors in the estimated parameters, ${\hat{T}}_{2_{ROI_{6}}}$ and ${\hat{f}}_{ROI_{6}}$, are plotted in (f) for all possible values of ΔTE. We observed good agreement between the reference signals and those disentangled with BSS.
**FIGURE S3** Separation of three compartments and parameter estimation for the phantom data. The *simulated* dataset was generated from the signal sources in (a). The *measured* datasets were calculated from the measured signals for ROI_5_ (b), ROI_6_ (c), and ROI_11_ (d). The mixed signals for both datasets (shown in (e)) show a mismatch due to measurement errors. They were disentangled with BSS, as shown in (f). We fixed TE_1_ = 77.5 ms and TE_3_ = 127.5 ms, and varied TE_2_ to minimize the condition number of **A** (shown in (g)). The relative errors of the estimated parameters are plotted for different values of the TE_2_ in (h).
**FIGURE S4** Simulated diffusion signals for IE and CSF. Synthetically generated diffusion signals for 30 directions (*b* = 1000 s/mm^2^) and one non‐diffusion weighted measurement. We modeled diffusion as a Gaussian process with MD of IE and CSF equal to $0.7\times 10^{-3}$ and $3\times 10^{-3}$ mm^2^/s respectively,^28^ and standard deviations of $0.3\times 10^{-3}$ and $0.1\times 10^{-3}$ mm^2^/s respectively to distinguish between hindered anisotropic (IE) and free isotropic (CSF) diffusivity.
**FIGURE S5** Convergence for two compartments (IE and CSF) with overlapping *T*_2_ constraints and no *S_CSF_* prior. This figure extends the analysis of Figure 2 for SNR = 100 and 150. The stability for *f_IE_* increases with SNR (a and b) and with *f_IE_* for $T_{2_{IE}}$ (c and d).
**FIGURE S6** Convergence for two compartments (IE and CSF) with non‐overlapping *T*_2_ constraints and *S_CSF_* prior. This figure extends the analysis of Figure 3 for SNR = 100 and 150. The size and stability of the convergence area for *f_IE_* and $T_{2_{IE}}$ increase with SNR.
**FIGURE S7** Convergence for two compartments (IE and CSF) with non‐overlapping *T*_2_ constraints and *S_CSF_* prior when only one is actually present in the tissue. This figure extends the analysis of Figure 4 for SNR = 100 and 150. The SNR does not play an important role in the definition of the convergence area.
**FIGURE S8** Convergence for two compartments (IC and EC) with overlapping *T*_2_ constraints and no other priors. This figure extends the analysis of Figure 5 for SNR = 100 and 150. The influence of SNR on *f* and $T_{2_{IC}}$ is small.
**FIGURE S9** Convergence for three compartments (myelin, IE, and CSF) with non‐overlapping *T*_2_ constraints and *S_CSF_* prior. This Figure extends the analysis of Figure 6 for SNR = 100 and 150.
**FIGURE S10** Convergence for two compartments (IE and CSF) with overlapping *T*_2_ constraints and *S_CSF_* prior. The mean and the standard error of *f_IE_* absolute error (a and b), and the mean and the standard error of $T_{2_{IE}}$ (c and d), and $T_{2_{CSF}}$ (e and f) relative error per unit (p.u.). Red and white lines mark the 0.2 and 0.1 contour respectively. One thousand simulations were run for each combination of SNR, *f_IE_*, $T_{2_{IE}}$, and ΔTE. $T_{2_{IE}}$ and $T_{2_{CSF}}$ were bound between 0–1000 ms and 0–3000 ms respectively. *S_CSF_* was set to have isotropic diffusivity with value $3\times 10^{-3}$ mm^2^/s. We defined the convergence area as the one with error lower than 0.1 for *f_IE_* and $T_{2_{IE}}$. Notice the growth of the converge area compared to the lack of priors (Figures 2 and S5).
**FIGURE S11** Convergence for two compartments (IE and CSF) with non‐overlapping *T*_2_ constrained and no *S_CSF_* prior. The mean and the standard error of *f_IE_* absolute error (a and b), and the mean and the standard error of $T_{2_{IE}}$ (c and d), and $T_{2_{CSF}}$ (e and f) relative error per unit (p.u.). Red and white lines mark the 0.2 and 0.1 contour respectively. One thousand simulations were run for each combination of SNR, *f_IE_*, $T_{2_{IE}}$, and ΔTE. $T_{2_{IE}}$ and $T_{2_{CSF}}$ were bound between 0–300 ms and 300–3000 ms respectively. No prior was imposed on *S_CSF_*. We defined the convergence area as the one with error lower than 0.1 for *f_IE_* and $T_{2_{IE}}$. Non‐overlapping *T*_2_ bounds stabilize the factorization, compared to Figures 2 and S5, although not as much as using priors on the signal sources (Figure S10).
**FIGURE S12** Convergence for two compartments (IE and CSF) with fixed $T_{2_{CSF}}$ and no *S_CSF_* prior. The mean and the standard error of *f_IE_* absolute error (a and b), and the mean and the standard error of $T_{2_{IE}}$ (c and d), and $T_{2_{CSF}}$ (e and f) relative error per unit (p.u.). Red and white lines mark the 0.2 and 0.1 contour respectively. One thousand simulations were run for each combination of SNR, *f_IE_*, $T_{2_{IE}}$, and ΔTE. $T_{2_{IE}}$ was bound between 0–300 and $T_{2_{CSF}}$ fixed to 2000 ms. No prior was imposed on *S_CSF_*. We defined the convergence area as the one with error lower than 0.1 for *f_IE_* and $T_{2_{IE}}$. Fixing the value of $T_{2_{CSF}}$ does not have any effect on the size of the convergence area, while bounding $T_{2_{IE}}$ does it (see Figure S11).
**FIGURE S13** Convergence for two compartments (IE and CSF) with non‐overlapping *T*_2_ constraints and *S_CSF_* prior. The mean and standard error of *f_IE_* absolute error (a and b), and mean and standard error of $T_{2_{IE}}$ (c and d), and $T_{2_{CSF}}$ (e and f) relative error per unit (p.u.). Red and white lines mark the 0.2 and 0.1 contour respectively. One thousand simulations were run for each combination of SNR, *f_IE_*, $T_{2_{IE}}$, and ΔTE. $T_{2_{IE}}$ and $T_{2_{CSF}}$ were bound between 0–300 ms and 300–3000 ms respectively. *S_CSF_* was set to have isotropic diffusivity with value $3\times 10^{-3}$ mm^2^/s. We defined the convergence area as the one with error lower than 0.1 for *f_IE_* and $T_{2_{IE}}$. Incorporating prior knowledge on the behavior of the signal sources (as CSF) improves convergence and stability more than bounding *T*_2_ (Compare with Figures S10 and S11)
**FIGURE S14** Simulated diffusion signals for intra and extra‐cellular water compartments. Synthetically generated diffusion signals for 30 directions (b = 1000 s/mm^2^) and one non‐diffusion weighted measurement. We modeled diffusion as a Gaussian process with MD of intra‐cellular (IC) and extra‐cellular (EC) equal to $0.6\times 10^{-3}$ and $0.8\times 10^{-3}$ mm^2^/s respectively (to keep the MD of parenchyma equals to $0.7\times 10^{-3}$ mm^2^/s)^28^ and standard deviations of $0.3\times 10^{-3}$ and $0.1\times 10^{-3}$ mm^2^/s respectively to distinguish between a more (IC) and less (EC) hindered anisotropic diffusivity.
**FIGURE S15** FA and MD of the BSS‐disentangled IE signal against the standard DTI and Pasternak's free‐water elimination (FWE) for subject one. Comparisons of the FA (b) and MD (d) histograms calculated from the separated IE signals are plotted against the standard DTI fit and Pasternak's method for the short TE measured data. MD (c) and colored FA (a) maps are also included for comparison. We observed a CSF correction effect in the long ΔTE BSS for FA in agreement with Pasternak's FWE. However, both method disagree for MD, where Pasternak's introduces spatial over‐regularization. See Figure 9 for subject two.
**FIGURE S16** Repeatability analysis showing intra‐subject variability. A healthy volunteer was scanned six times. The FA (a) and MD (b) histograms for standard DTI, BSS and Pasternak's method are shown. These histograms were fragmented in sectors and the relative changes in number of voxels per sector and repetition for BSS and Pasternak's methods were computed. Statistical t‐tests were run per sector to determine the level of significance of the differences between BSS and Pasternak's results (d and e). BSS and FLAIR $T_{2_{IE}}$ histograms (c) showed good agreement. Their peak and the full width half maximum (FWHM) were used for t‐test comparison between BSS and FLAIR (f) highlighting the concordance.
**FIGURE S17** Reproducibility analysis showing inter‐subject variability. Twenty healthy volunteers were scanned. The FA (a) and MD (b) histograms for standard DTI, BSS and Pasternak's method are shown. These histograms were fragmented in sectors and the relative changes in number of voxels per sector and repetition for BSS and Pasternak's methods were computed. Statistical t‐tests were run per sector to determine the level of significance of the differences between BSS and Pasternak's results (d and e). Notice that the inter‐subject variability is larger than intra‐subject (Figure S16). BSS and FLAIR $T_{2_{IE}}$ histograms (c) were depicted. Their peak and the full width half maximum (FWHM) were used for t‐test comparison between BSS and FLAIR (f).
**Table S1** Phantom reference values and BSS estimates. The ROIs in the phantom experiment was built using the concentrations of agar and sucrose shown here. Signal decays along the diffusion dimension were compared to each other to ensure that they were all different, as required by BSS (see supplementary Figure S18). For reference, the *T*_2_ values were characterized using an NNLS fit. Confidence intervals were taken at the half maxima of the NNLS spectral peaks. In addition, a second method, EASI‐SM,^17^ was used to confirm the validity of the fits. Finally, the $T_{2_{BSS}}$ values were estimated for ΔTE = 50 ms and compared with the NNLS and EASI‐SM references (where *ϵ* refers to the relative error).
