# Supplementary material for: A diffusion model‐free framework with echo time dependence for free‐water elimination and brain tissue microstructure characterization
Source: Magn Reson Med. 2018 Mar 23;80(5):2155–72. doi: 10.1002/mrm.27181 (PMC6790970; doi:10.1002/mrm.27181)
Supplement: Supplementary file 1 — FIGURE S1 Evolution of the relative error in the T 2 estimate with ΔTE for one compartment. The mean relative error of T 2 estimated using BSS is shown in (a) for NNLS and in (b) for EASI‐SM references. ΔTE goes from 5 ms (darker colors) to 50 ms (lighter colors). The dependence of T 2 on the direction (slope) of the columns of A (Equation 3) is shown in (c), where it can be seen how increasing ΔTE improves the dynamic range of the slope of A, resulting in a better estimate for T 2. Except for ROI1 and ROI11, the remaining ones reduce the T 2 mean relative error as ΔTE increases (a and b, lighter colors are closer to zero), in agreement with plot c. FIGURE S2 Separation of two compartments and parameter estimation for the phantom data. The signal sources of the simulated dataset are plotted in (a), and the measured data generated from the sources in (b). The resulting mixtures for both datasets are shown in (c). We use the subscripts M and S to refer to estimates for the measured and simulated datasets, respectively. Measurement errors are highlighted by the differences between the measured and simulated signals, shown in (c). BSS disentangled the original sources for both datasets, as shown in (d). We chose a ΔTE of 50 ms to minimize the condition of A (shown in (e)) and increase the numerical stability of the framework. Finally, the relative errors in the estimated parameters, T^2ROI6 and f^ROI6, are plotted in (f) for all possible values of ΔTE. We observed good agreement between the reference signals and those disentangled with BSS. FIGURE S3 Separation of three compartments and parameter estimation for the phantom data. The simulated dataset was generated from the signal sources in (a). The measured datasets were calculated from the measured signals for ROI5 (b), ROI6 (c), and ROI11 (d). The mixed signals for both datasets (shown in (e)) show a mismatch due to measurement errors. They were disentangled with BSS, as shown in (f). We fixed TE1 = 77.5 ms and TE3 = [file MRM-80-2155-s001.pdf]

Supporting Material

Phantom experiment

Methods

We built a phantom based on pure water and eleven different concentrations of agar and sucrose, producing eleven unique combinations of T_2 and diffusivity (Table S1) (62). We scanned the phantom (see below) and defined regions of interest (ROIs) in the tubes containing the eleven concentrations. Each ROI was independently processed with BSS to study the one compartment case. We also mixed the signals from two ROIs to generate a pair of two-compartment datasets and fed these to our BSS solver. Finally, for the three-compartment case we combined three ROIs and separated them with BSS. We were aiming to demonstrate that our framework was able to yield T_2 estimates for one compartment; and volume fraction, T_2 estimates and diffusion signal separation for two and three compartments.

For reference, we measured multi-echo SE acquisitions (Signa HDx 3T, GE Healthcare, Milwaukee, WI) for TE values from 10–640 ms in 10 ms increments. The following values were constant: TR = 3460 ms; NEX = 2; 128 × 128 matrix size; FOV = 240 mm; and 7 mm slice thickness. Eleven diffusion experiments were undertaken for TE values from 77.5–127.5 ms in 5 ms increments. the following parameters were constant: FOV = 240 mm; 7 mm slice thickness; 64 × 64 matrix size; TR = 4000 ms; ASSET = 2; A/P diffusion direction; and 41 equally spaced b-values from 0–2000 s/mm^2.

The multi-echo SE signals were averaged within each ROI. Each signal was fitted with NNLS (13) using a log-scaled grid with T_2 values at 500 points between 10–2000 ms. We used the maximum values of the NNLS T_2 spectra as ROI reference values (Figure S1) and fitted the signal from each ROI with EASI-SM (17) for reassurance.

One compartment

For one compartment ($M = 1$), we processed the diffusion data from ten pairs of TE measurements ($N = 2$) with BSS to include the relaxation effects in the dataset. For each pair, the short TE was fixed at 77.5 ms, while the long TE was increased from 82.5–127.5 ms along with the measured echo times. We constrained the solution space for the estimated $\hat{T}_2$ values to 10–2000 ms to account for all the ROIs. No other prior information was considered. We report the evolution of the T_2

values estimated using BSS for each ROI and the differences between the short and long TEs (ΔTE) compared with their reference values (Figure S1).

Two compartments

For two compartments ($M = N = 2$), we created two different datasets. First, we used the diffusion data measured at the shortest TE for ROI_6 and ROI_{11} as the sources, $\mathbf{S}$. These signals did not contain relaxation information (Figure S2a). Thus, to mix them together, we had to compute the mixing matrix ($\mathbf{A}$) as in in Eq. 2. We used their reference T_2 values, the experimental TEs, and a volume fraction of $f_{ROI_6} = 0.7$ (Figure S2c). We called this the *simulated* dataset, given that the signals were mixed under ideal conditions. Second, we normalized the measured data for each ROI and TE to its maximum value at the shortest TE to allow for later comparison of the volume fractions. In this case, the signals already contained the relaxation information (Figure S2b), so we did not need to compute $\mathbf{A}$. We scaled the normalized measured signals using the given volume fraction and added them together to create the mixed measurements, $\mathbf{X}$. We called this the *measured* dataset (Figure S2c). It accounts for system imperfections like signal drift, imperfect non-diffusion weighting, and eddy currents. To constrain the solution of the cALS algorithm we used $T_{2_{ROI_{11}}}$ and $S_{ROI_{11}}$ as the prior knowledge and searched for $\hat{T}_{2_{ROI_6}}$ between 0–200 ms.

Three compartments

We extended the two-compartments experiment to three ($M = N = 3$) by adding ROI_5. *Simulated* and *measured* datasets were created as for the two-compartments case (Figures S3a, S3b, S3c, S3d and S3e). This time, we used the volume fractions $f_{ROI_5} = 0.2$ and $f_{ROI_6} = 0.6$. To limit the solution space of the cALS algorithm, we assumed $T_{2_{ROI_{11}}}$ and $S_{ROI_{11}}$ to be prior the knowledge. We also constrained the $\hat{T}_{2_{ROI_5}}$ and $\hat{T}_{2_{ROI_6}}$ values to be between 0–50 ms, and 50–200 ms, respectively. For the two- and three-compartment experiments we report the stability of the framework, the relative error of the parameters and the disentangling capability of the method.

Results

One compartment

There was a correlation between the estimated T_2 values for one compartment obtained using multi-echo SE for 17 TEs and BSS for 2 TEs (Figures S1a, S1b, and Table S1). The T_2 estimates from

ROI_2 to ROI_{10} showed relative errors below 0.1 p.u. for a ΔTE of 50 ms (Figures S1a and S1b). The decreasing error trend is due to the relationship between the slope of a column of $\mathbf{A}$ and its T_2 value (Figure S1c). As ΔTE increased, the dynamic range of the slope of $\mathbf{A}$'s columns expanded, yielding better T_2 estimates. On the other hand, in Figures S1a and S1b, ROI_1 and ROI_{11} showed increasing errors as ΔTE increased. In the case of ROI_1, this was due to the low SNRs of the measurements at the experimental TEs. The noise floor caused changes in the signals for longer TEs that biased the T_2 estimates. The effect observed in ROI_{11} cannot be explained by SNR or T_2-slope dependence. We attribute this result to an underestimation of the reference T_2 value due to incomplete recovery of the longitudinal magnetization, which is caused by the short experimental TR (TR = 3460 ms) compared to the T_1 value of ROI_{11} ($T_{1_{11}}$ = 2200 ms). Finally, the error between the NNLS and BSS T_2 estimate for ROI_4, ROI_6, ROI_7, and ROI_8 is larger than for the others (Figure S1a) at ΔTE = 50 ms, except for ROI_1 and ROI_{11} already discussed. For these ROIs, NNLS converges to a bi-exponential decay (See Figure S19 and Table S1) increasing the value of the long T_2 coefficient compared to BSS and EASI-SM.

Two compartments

The disentangled signals for the *simulated* dataset replicated the profiles of the reference sources (Figure S2d). Moreover, the maximum relative errors for $\hat{f}_{S,ROI_6}$ and $\hat{T}_{2_{S,ROI_6}}$ were below 0.01 p.u. for all the possible ΔTE values. Interestingly, BSS was also able to separate the signal sources of the *measured* dataset (Figure S2d). This data accounted for non-ideal conditions due to system imperfections, such as signal drift, eddy currents, or imperfect non-diffusion weighting (Figure S2b, S2c, S2d, and S2f). In that case, the relative error in the $\hat{T}_{M_{S,ROI_6}}$ estimate remained under 0.1 p.u. for all ΔTEs above 10 ms. We believe that the 0.15 p.u. error in $\hat{f}_{M,ROI_6}$ is due to the differences between the *simulated* and *measured* signals at b = 0 s/mm^2, their influence on Eq. 4, and propagation of the error in the $\hat{T}_2$ estimate. Finally, we also observed a small stabilization effect in the volume fraction estimates as ΔTE increased (Figure S2f). This behavior is due to reductions in $\mathbf{A}$'s condition number improving the cALS algorithm's numerical stability (Figure S2e).

Three compartments

The condition number of $\mathbf{A}$ significantly increased compared with the two-compartment model (Figures S2e and S3g). Results for the *simulated* data (Figures S3a, S3e and S3f) showed that the signals for compartments ROI_6 and ROI_{11} had been separated, in agreement with their references.

Likewise, the relative errors in the $\hat{T}_{2_{S,ROI_6}}$ and $\hat{f}_{S,ROI_6}$ estimates were below 0.01 p.u., confirming the good separation. It is worth noting that the signal for the fast-decaying compartment (ROI_5) was detected, despite being heavily contaminated by the ROI_6. We believe that this result is due to the comparably large experimental TE, reducing its contribution to the observed signal. Equivalently, we found a 0.15 p.u. error in the $\hat{f}_{S,ROI_5}$ estimate and 0.45 p.u. in the $\hat{T}_{2_{S,ROI_5}}$ estimate.

Results for the *measured* data when **A**'s condition number was lowest showed that the signals from ROI_6 and ROI_{11} had still been separated, in agreement with the references (Figure S3f). However, the signal from ROI_5 was lost due to acquisition imperfections, bad conditioning of **A**, and small contributions of this compartment at the measured TEs. On the other hand, the $\hat{T}_{2_{M,ROI_6}}$ estimate was stability with a relative error of 11%. In contrast, $\hat{f}_{M,ROI_6}$ was more unstable due to the bad conditioning of the system and propagation of the error in the $\hat{T}_{2_{M,ROI_6}}$ estimate.

Repeatability and reproducibility

Following the simulations, phantom experiment, and in vivo studies for incremental ΔTE, repeatability and reproducibility analyses were conducted to demonstrate the stability and reliability of our method. In this supporting section we described the experimental setup and results, while the discussion remained in the main body of the paper.

Methods

Repeatability

A healthy volunteer (male, 28 years old) was scanned six times in a 3.0T GE MR750w scanner (GE Healthcare, Milwaukee, WI). For each repetition we acquired two diffusion PGSE EPI volumes with TE values 75.3 and 135.3 ms (ΔTE = 60 ms); FOV = 225 mm; 4 mm slice thickness; 22 slices; TR = 8000 ms; 96 $\times$ 96 matrix size; ASSET = 2; 30 directions; and one non-diffusion-weighted volume. Besides, one non-diffusion-weighted volume was acquired with reversed polarity at each TE. Finally, a FLAIR multi-echo sequence was acquired with the same geometrical prescription for TE = 20 − 260 ms in 30 ms increments; ASSET = 0; and TR = 8000 ms. An extra volume was acquired with reverse polarity at TE = 20 ms.

Diffusion and FLAIR data were processed with FSL Topup (63, 64) and Eddy (65) to correct for distortions. The long TE diffusion volume was registered to the short TE one with FLIRT and processed with BSS for two compartments (IE and CSF). We used literature CSF values ($T_{2_{CSF}}$ = 2

s and $D_{CSF} = 3 \times 10^{-3}$ mm^2/s) as the prior knowledge, and constrained $T_{2_{IE}}$ between 0 – 200 ms. Then, the resulting tissue volume was fitted to the DTI model using standard linear regression (FSL FDT). For comparison, the distortion free short TE diffusion volume was also fitted to the DTI model and free-water corrected with Pasternak's method. Finally, FLAIR data were matched to a dictionary of mono-exponential decays from 0 – 300 ms with 1 ms increments.

We reported the FA and MD histograms for the six repetitions of the standard DTI fit for the short TE, IE BSS, and Pasternak's method (Fig S16a and b). The free-water correction effects were quantified dividing the histograms in sectors, and computing relative change per sector in the number of voxels of BSS and Pasternak's method reference to the standard DTI fitting (Fig S16d and e). FA histograms were split in four quarters, while MD in two sectors with threshold in the IE literature value (MD $= 0.7 \times 10^{-3}$ mm^2/s) (28). Statistical t-test analyses were conducted to determine the differences between BSS and Pasternak's FWE. Histograms of BSS $T_{2_{IE}}$ and FLAIR T_2 (Fig S16c) were compared by their peak and full width half maximum (FWHM) values (Fig S16f).

Reproducibility

Twenty healthy volunteers (8 females, 26 years old in average) were scanned in a 3.0T GE MR750 scanner (GE Healthcare, Milwaukee, WI) at the Max Planck Institute of Psychiatry in Munich, Germany. Two diffusion PGSE EPI volumes with TE values 60.1 and 120.1 ms (ΔTE $= 60$ ms) with TR $= 5000$ ms were acquired. All the other acquisition parameters and data processing steps were as described for the repeatability experiment. Due to scanner availability FLAIR data was only acquired for half of the subjects. Histograms of FA, MD, and T_2, along with their statistical analyses were reported in Figure S17.

Results

Repeatability

The histograms of FA, MD and T_2 (Figure S16a, b, and c) showed highly overlapping curves for each repetition and method, denoting good repeatability for all of them. After splitting the FA histogram in four sectors and computing the relative change in the area per sector for BSS and Pasternak's method (Figure S16d), we found that the lowest ratio between the mean and the standard deviation for BSS was 5.3 (sector IV) and 11.4 for Pasternak's (sector II). The fact that the mean is 5.3 larger

than the standard deviation suggested that BSS produces highly stable free-water correction. This result held also for MD, where we found ratios of 9.9 and 65.4 for BSS and Pasternak's method in sector II; and T_2, with mean to standard deviation ratios of 62.3 and 10.4 in the peak and FWHM values for BSS.

Interestingly, we found that BSS and Pasternak's free-water correction yield statistically different results for FA > 0.25 (sectors II, III, and IV, Figure S16d and e) and both MD sectors, with a significance level $\alpha = 0.01$. Finally, BSS $T_{2_{IE}}$ and FLAIR $T_{2_{IE}}$ histogram peaks were not statistically different, while the FWHM values were with a small effect. These findings indicate a good agreement of BSS with the FLAIR reference (Figure S16f).

Reproducibility

The FA, MD and T_2 histograms showed larger inter-subject variability (Figure S17a, b, and c) compared to the intra-subject one (Figure S16a, b, and c)) . The lowest ratios between the mean and the standard deviation of the free-water correction factor were 3.9 for BSS FA (sector IV) and 6.8 for Pasternak's FA (sector II); and 4.9 for BSS MD (sector II) and 20.5 for Pasternak's MD (sector II). Furthermore, mean to standard deviation ratios of BSS $T_{2_{IE}}$ were 47.2 and 8.9 for peak and FWHM values. These results suggested that BSS experiments are highly reproducible among subjects.

The statistical differences found in the repeatability study in FA sectors III and IV, and both MD sectors were still present in this analysis, indicating consistent differences between BSS and Pasternak's method (Figure S17d and e).

We found a statistically significant difference ($\alpha = 0.01$) between the means of the histogram peaks of the BSS and FLAIR $T_{2_{IE}}$ but with a small size effect (Figure S17f). Which indicates that BSS might yield a small bias in group comparisons compared to FLAIR multi-echo.

Supporting Figures (for publication)

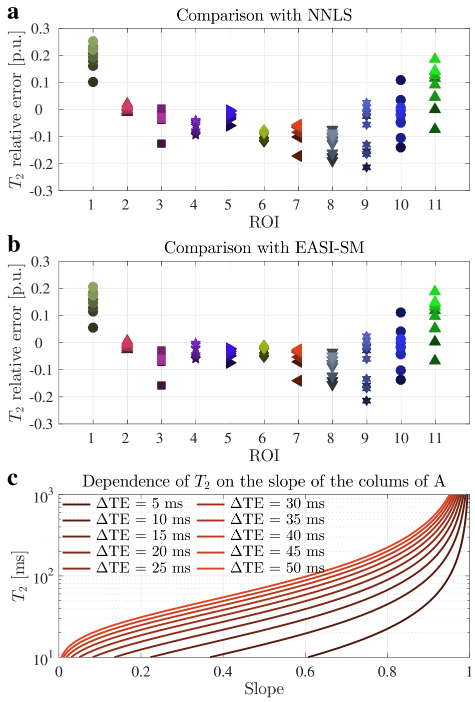

Figure S1: **Evolution of the relative error in the T_2 estimate with ΔTE for one compartment.**

The mean relative error of T_2 estimated using BSS is shown in (a) for NNLS and in (b) for EASI-SM references. ΔTE goes from 5 ms (darker colors) to 50 ms (lighter colors). The dependence of T_2 on the direction (slope) of the columns of **A** (Eq. 3) is shown in (c), where it can be seen how increasing ΔTE improves the dynamic range of the slope of **A**, resulting in a better estimate for T_2. Except for ROI_1 and ROI_{11}, the remaining ones reduce the T_2 mean relative error as ΔTE increases (a and b, lighter colors are closer to zero), in agreement with plot c.

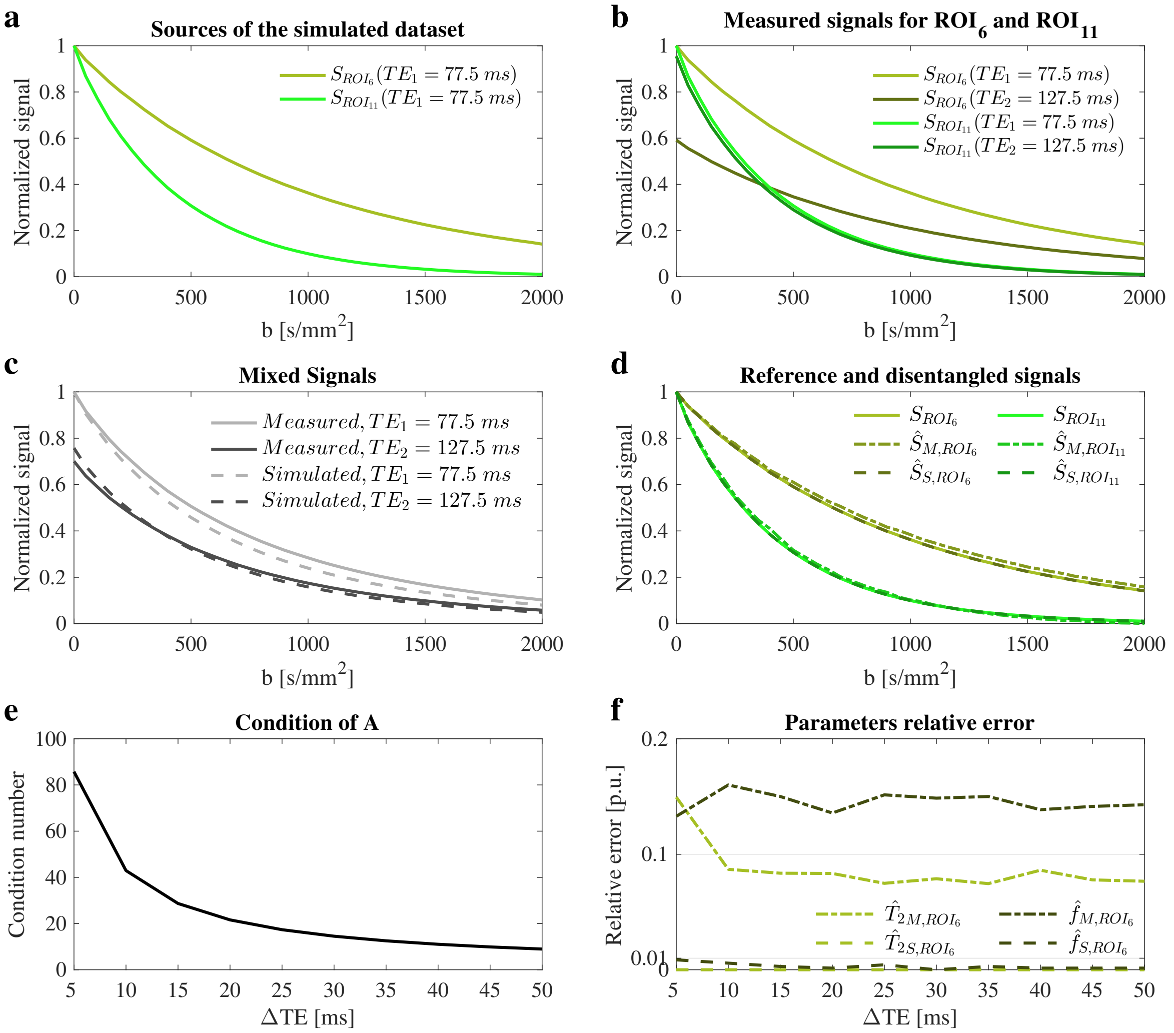

Figure S2: **Separation of two compartments and parameter estimation for the phantom data.**

The signal sources of the *simulated* dataset are plotted in (a), and the *measured* data generated from the sources in (b). The resulting mixtures for both datasets are shown in (c). We use the subscripts M and S to refer to estimates for the *measured* and *simulated* datasets, respectively. Measurement errors are highlighted by the differences between the *measured* and *simulated* signals, shown in (c). BSS disentangled the original sources for both datasets, as shown in (d). We chose a ΔTE of 50 ms to minimize the condition of $\mathbf{A}$ (shown in (e)) and increase the numerical stability of the framework. Finally, the relative errors in the estimated parameters, $\hat{T}_{2_{ROI_6}}$ and $\hat{f}_{ROI_6}$, are plotted in (f) for all possible values of ΔTE. We observed good agreement between the reference signals and those disentangled with BSS.

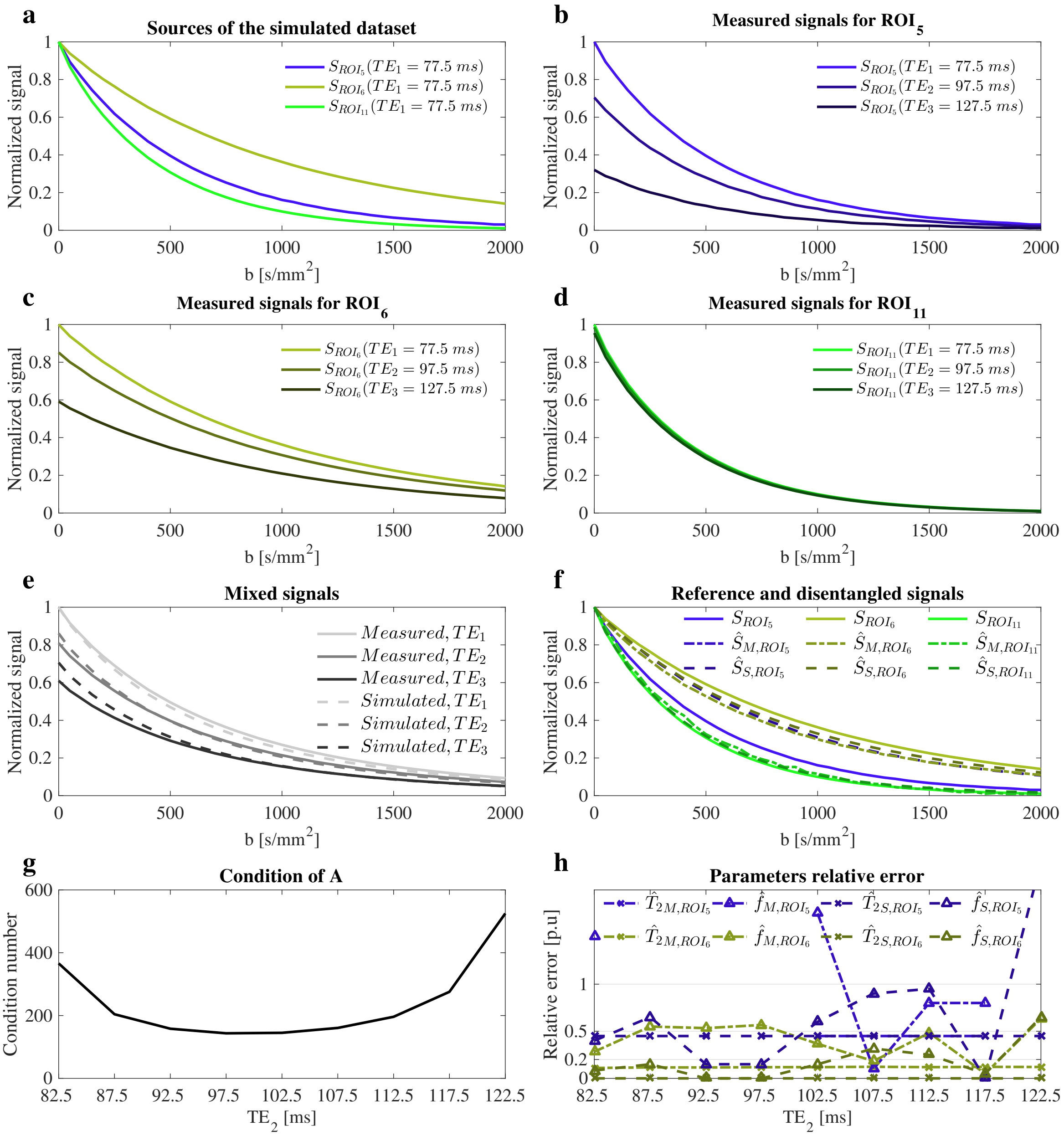

Figure S3: **Separation of three compartments and parameter estimation for the phantom data.**

The *simulated* dataset was generated from the signal sources in (a). The *measured* datasets were calculated from the measured signals for ROI$_5$ (b), ROI$_6$ (c), and ROI$_{11}$ (d). The mixed signals for both datasets (shown in (e)) show a mismatch due to measurement errors. They were disentangled with BSS, as shown in (f). We fixed TE$_1$ = 77.5 ms and TE$_3$ = 127.5 ms, and varied TE$_2$ to minimize the condition number of $\mathbf{A}$ (shown in (g)). The relative errors of the estimated parameters are plotted for different values of the TE$_2$ in (h).

ROI	Agar [%]	Sucrose [%]	$T_{2_{EASI-SM}}[ms]$	$T_{2_{NNLS}}$ [ms]	$T_{2_{BSS}}$ [ms]	ϵ_{NNSL} [%]	$\epsilon_{EASI-SM}$ [%]
1	5	15	25.02	23.88 ± 1.92	29.9	25.37	19.61
2	5	5	31.59	31.13 ± 2.19	31.6	1.43	0.04
3	3	30	37.68	36.50 ± 3.04	35.4	2.95	5.99
4	3	15	106.23	110.07 ± 7.93	106.0	3.70	0.22
5	3	5	45.40	44.66 ± 2.85	44.5	0.40	2.02
6	1	30	95.46	102.19 ± 10.30	93.9	8.13	1.66
7	1	15	222.22	228.94 ± 12.15	216.3	5.53	2.67
8	1	5	225.19	233.85 ± 13.84	213.4	8.76	5.25
9	0	30	457.08	456.37 ± 26.50	467.6	2.47	2.31
10	0	15	395.95	397.56 ± 21.17	401.0	0.87	1.28
11	0	0.5	876.97	881.23 ± 64.07	1008.6	14.46	15.01

Table S1: **Phantom reference values and BSS estimates.**

The ROIs in the phantom experiment was built using the concentrations of agar and sucrose shown here. Signal decays along the diffusion dimension were compared to each other to ensure that they were all different, as required by BSS (see supplementary Figure S18). For reference, the T_2 values were characterized using an NNLS fit. Confidence intervals were taken at the half maxima of the NNLS spectral peaks. In addition, a second method, EASI-SM (17), was used to confirm the validity of the fits. Finally, the $T_{2_{BSS}}$ values were estimated for ΔTE = 50 ms and compared with the NNLS and EASI-SM references (where ϵ refers to the relative error).

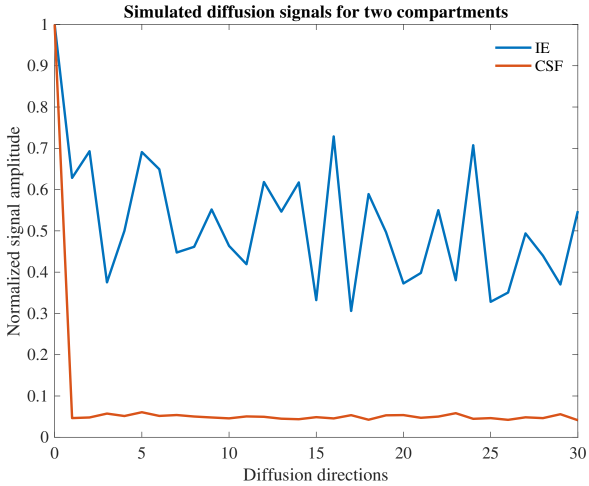

Figure S4: **Simulated diffusion signals for IE and CSF.**

Synthetically generated diffusion signals for 30 directions (b = 1000 s/mm^2) and one non-diffusion weighted measurement. We modeled diffusion as a Gaussian process with MD of IE and CSF equal to 0.7×10^{-3} and 3×10^{-3} mm^2/s respectively (28), and standard deviations of 0.3×10^{-3} and 0.1×10^{-3} mm^2/s respectively to distinguish between hindered anisotropic (IE) and free isotropic (CSF) diffusivity.

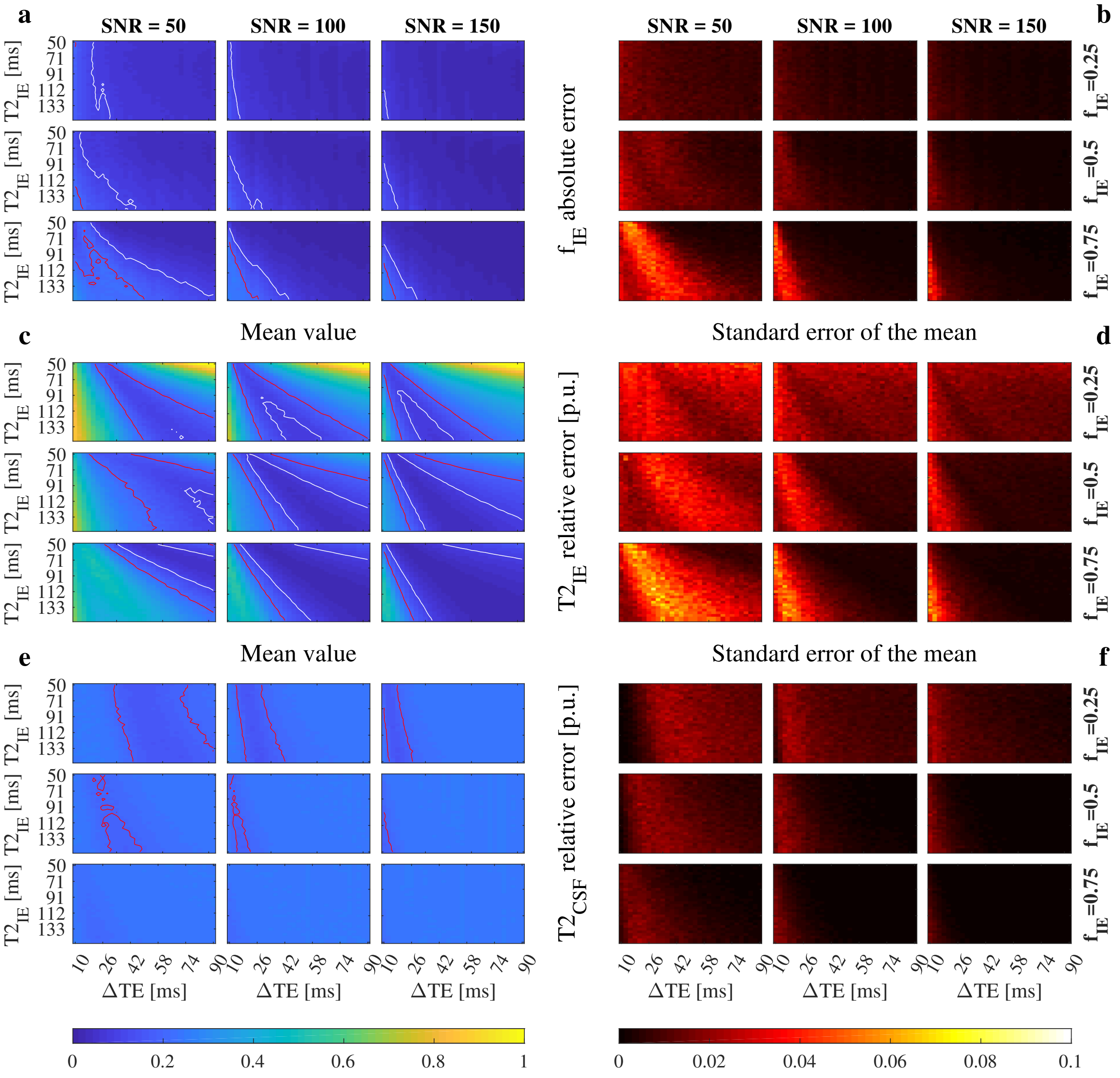

Figure S5: **Convergence for two compartments (IE and CSF) with overlapping T_2 constraints and no S_{CSF} prior.**

This figure extends the analysis of Figure 2 for SNR = 100 and 150. The stability for f_{IE} increases with SNR (a and b) and with f_{IE} for $T_{2_{IE}}$ (c and d).

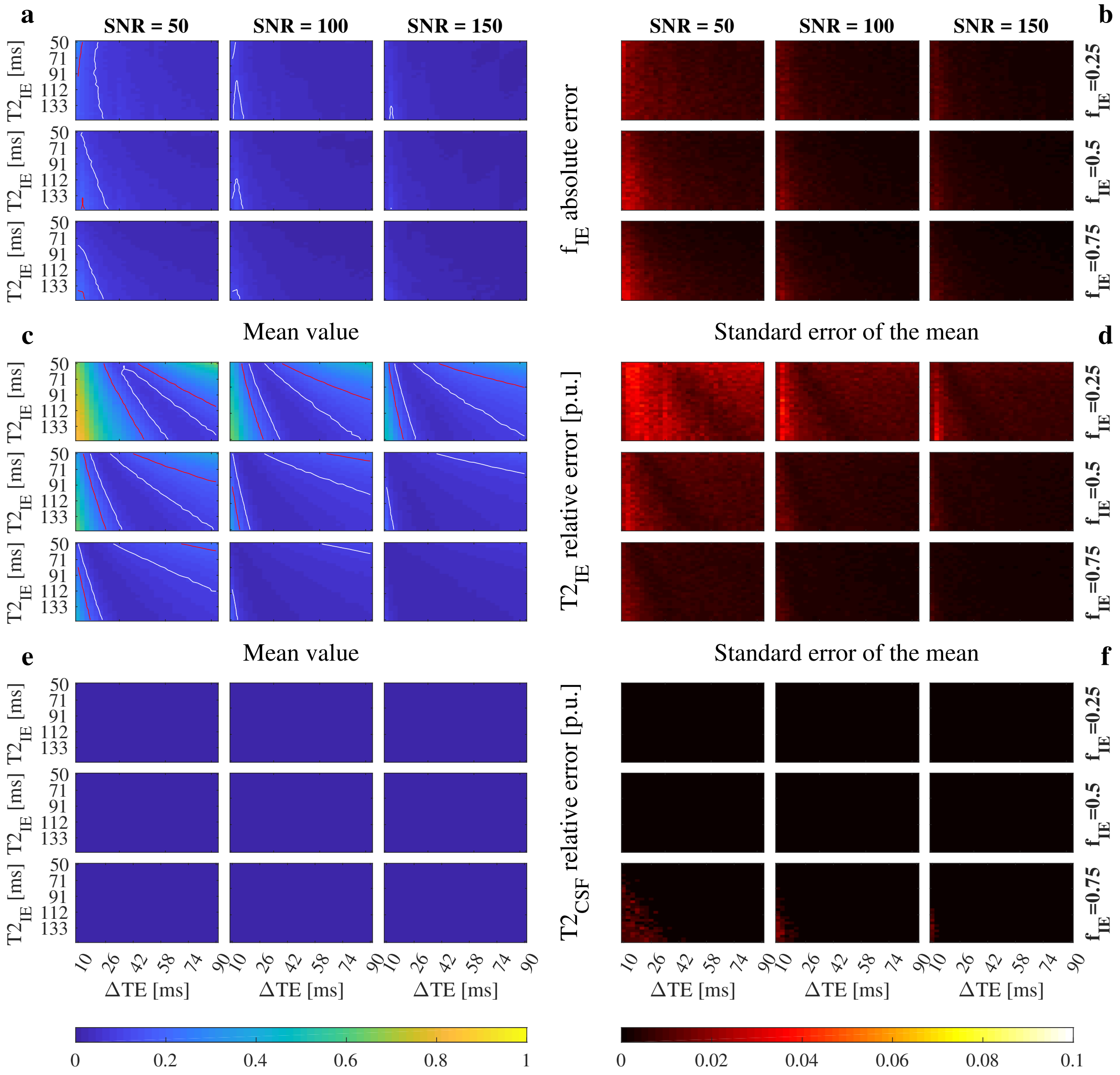

Figure S6: **Convergence for two compartments (IE and CSF) with non-overlapping T_2 constraints and S_{CSF} prior.**

This figure extends the analysis of Figure 3 for SNR $=$ 100 and 150. The size and stability of the convergence area for f_{IE} and $T_{2_{IE}}$ increase with SNR.

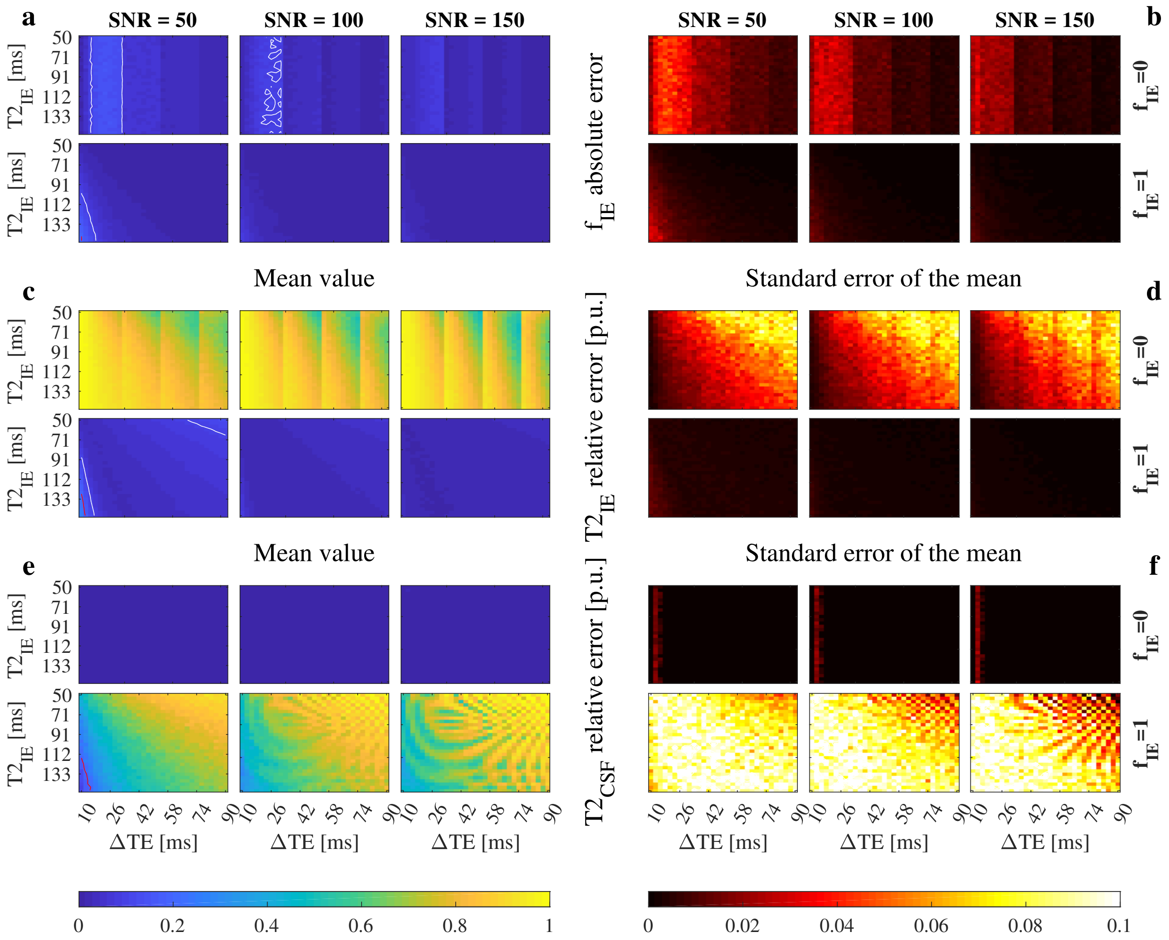

Figure S7: **Convergence for two compartments (IE and CSF) with non-overlapping T_2 constraints and S_{CSF} prior when only one is actually present in the tissue.** This figure extends the analysis of Figure 4 for SNR = 100 and 150. The SNR does not play an important role in the definition of the convergence area.

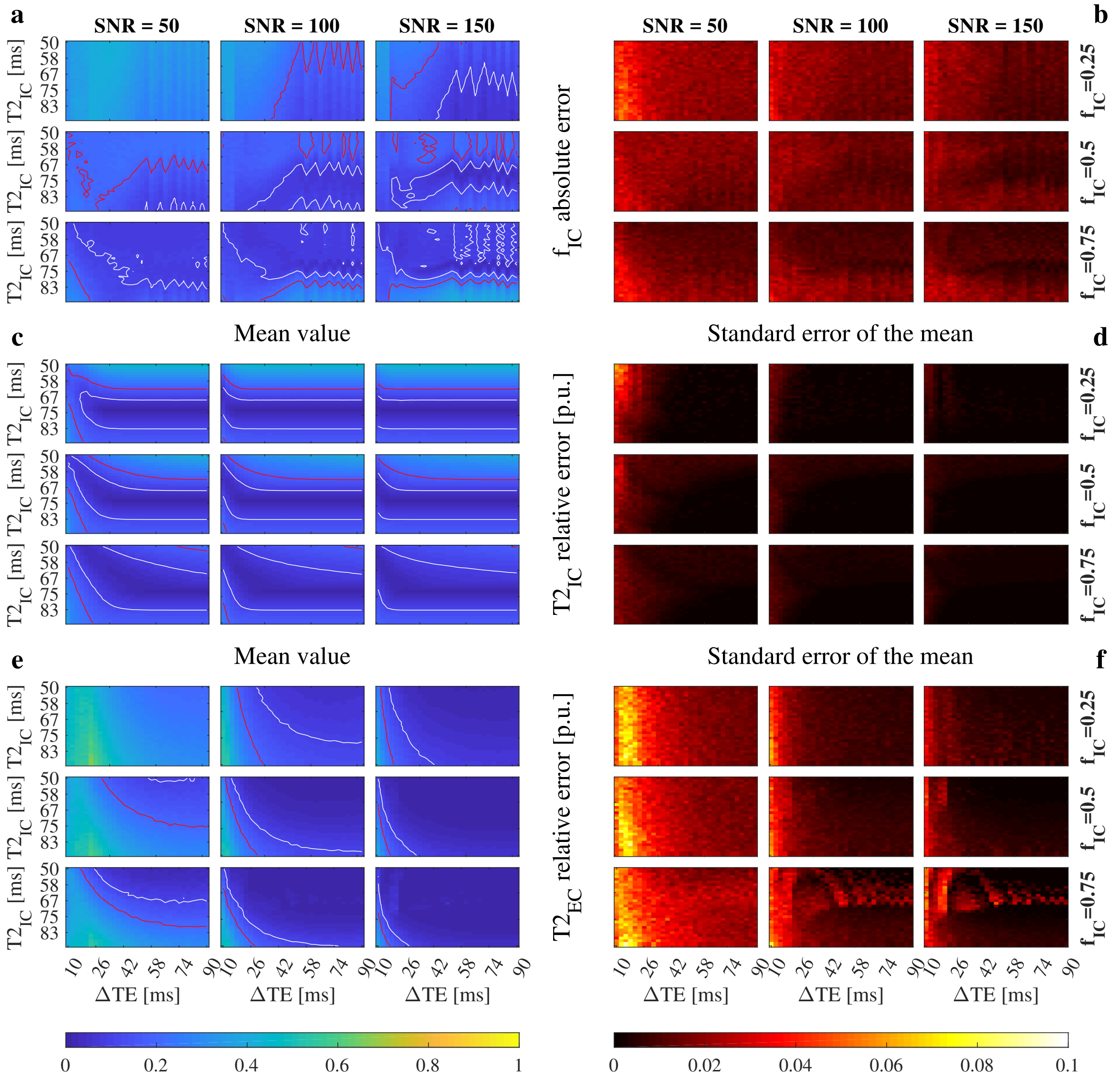

Figure S8: **Convergence for two compartments (IC and EC) with overlapping T_2 constraints and no other priors.**

This figure extends the analysis of Figure 5 for SNR = 100 and 150. The influence of SNR on f and $T_{2_{IC}}$ is small.

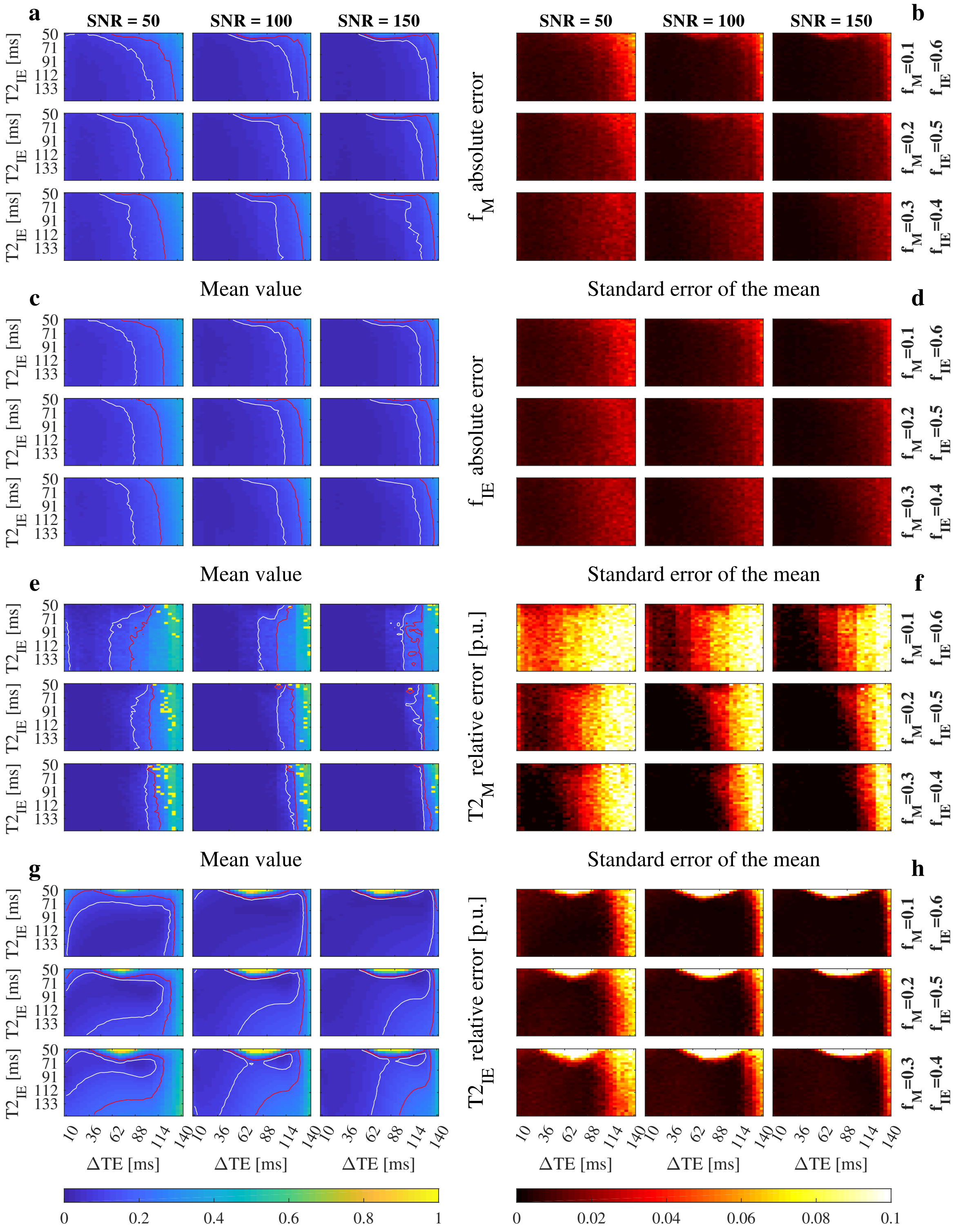

Figure S9: **Convergence for three compartments (myelin, IE, and CSF) with non-overlapping T_2 constraints and S_{CSF} prior.**

This Figure extends the analysis of Figure 6 for SNR $=$ 100 and 150.

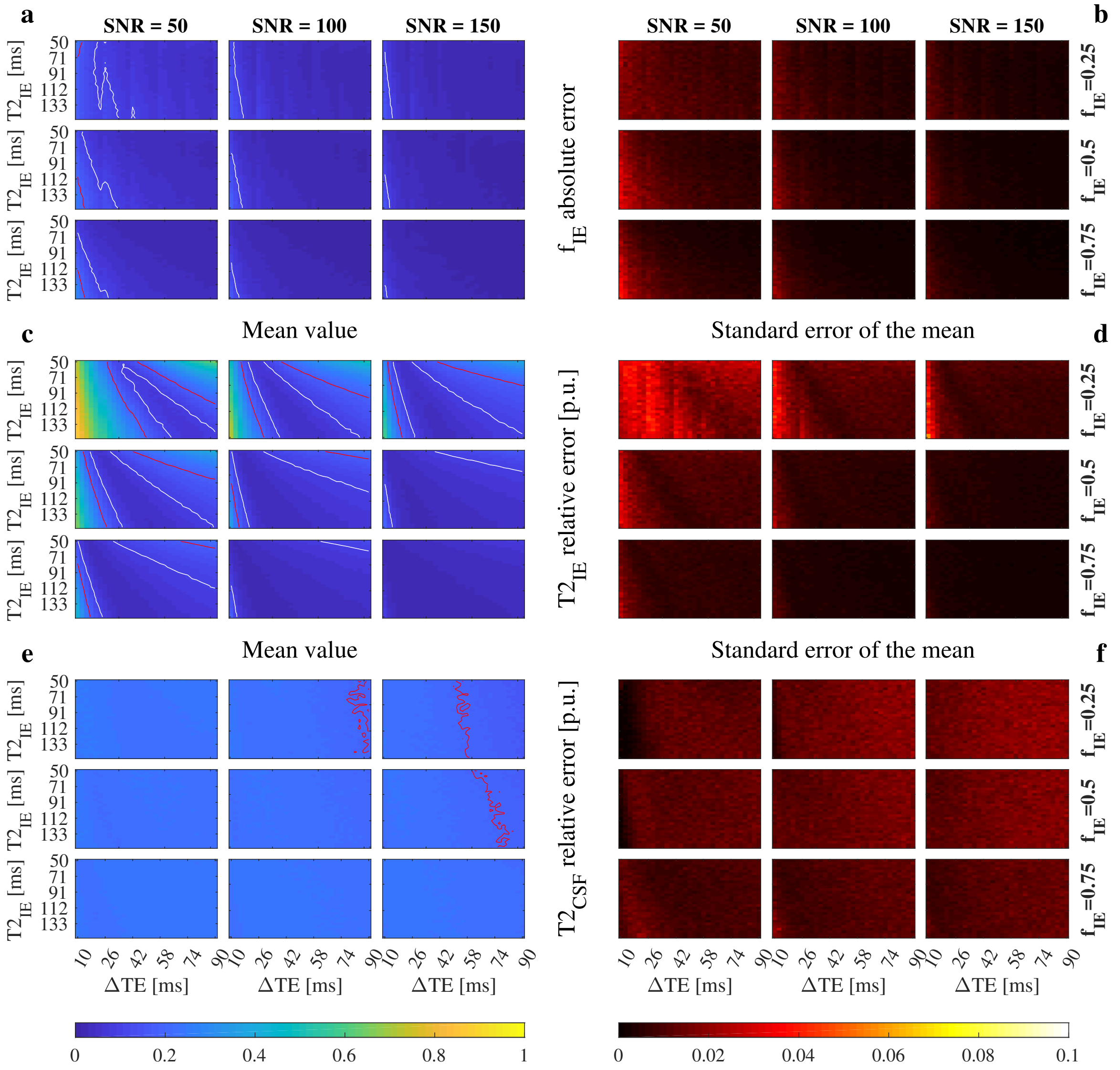

Figure S10: **Convergence for two compartments (IE and CSF) with overlapping T_2 constraints and S_{CSF} prior.**

The mean and the standard error of f_{IE} absolute error (a and b), and the mean and the standard error of $T_{2_{IE}}$ (c and d), and $T_{2_{CSF}}$ (e and f) relative error per unit (p.u.). Red and white lines mark the 0.2 and 0.1 contour respectively. One thousand simulations were run for each combination of SNR, f_{IE}, $T_{2_{IE}}$, and ΔTE. $T_{2_{IE}}$ and $T_{2_{CSF}}$ were bound between 0–1000 ms and 0–3000 ms respectively. S_{CSF} was set to have isotropic diffusivity with value 3×10^{-3} mm^2/s. We defined the convergence area as the one with error lower than 0.1 for f_{IE} and $T_{2_{IE}}$. Notice the growth of the converge area compared to the lack of priors (Figures 2 and S5).

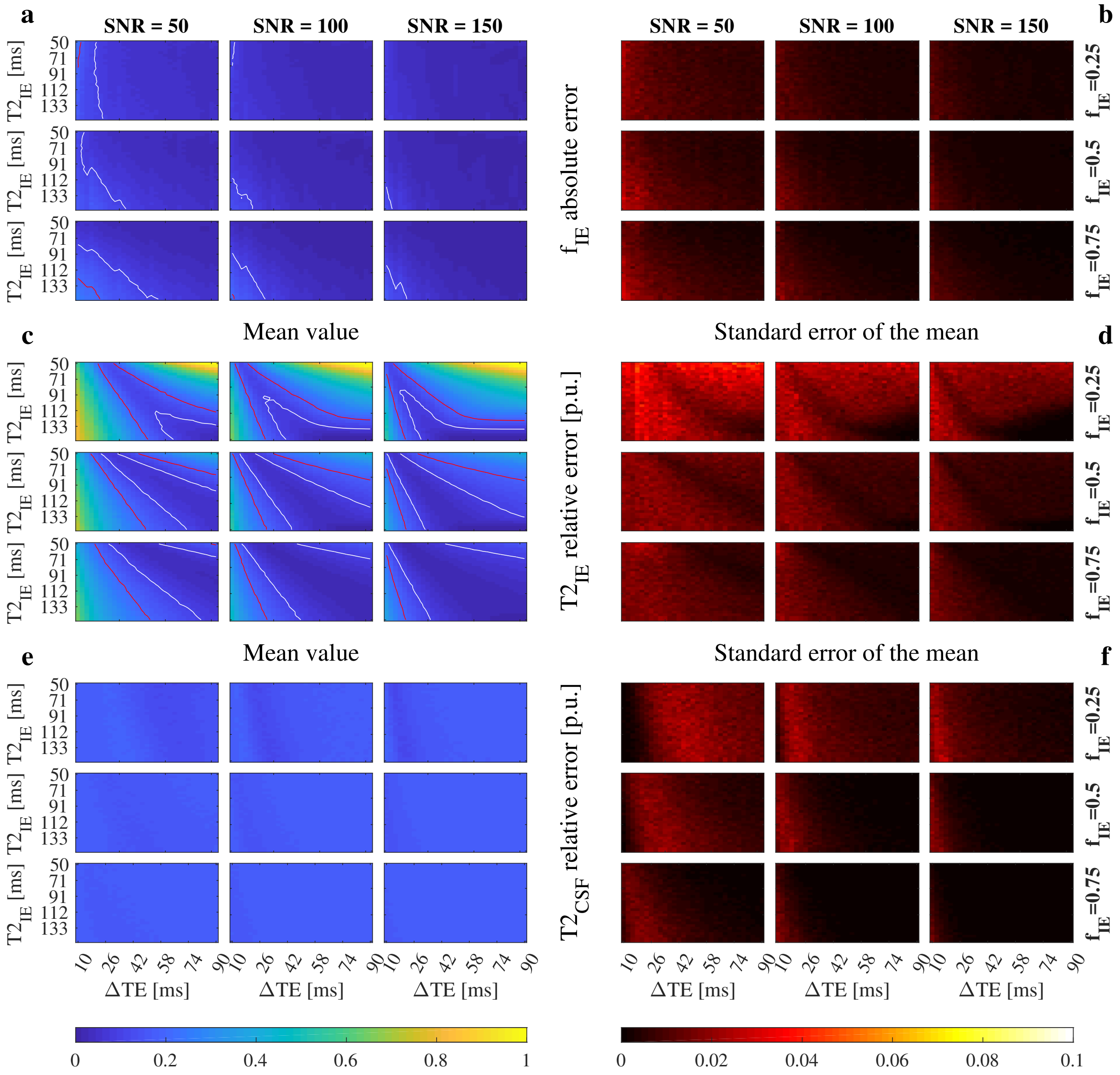

Figure S11: **Convergence for two compartments (IE and CSF) with non-overlapping T_2 constrained and no S_{CSF} prior.**

The mean and the standard error of f_{IE} absolute error (a and b), and the mean and the standard error of $T_{2_{IE}}$ (c and d), and $T_{2_{CSF}}$ (e and f) relative error per unit (p.u.). Red and white lines mark the 0.2 and 0.1 contour respectively. One thousand simulations were run for each combination of SNR, f_{IE}, $T_{2_{IE}}$, and ΔTE. $T_{2_{IE}}$ and $T_{2_{CSF}}$ were bound between 0–300 ms and 300–3000 ms respectively. No prior was imposed on S_{CSF}. We defined the convergence area as the one with error lower than 0.1 for f_{IE} and $T_{2_{IE}}$. Non-overlapping T_2 bounds stabilize the factorization, compared to Figures 2 and S5, although not as much as using priors on the signal sources (Figure S10).

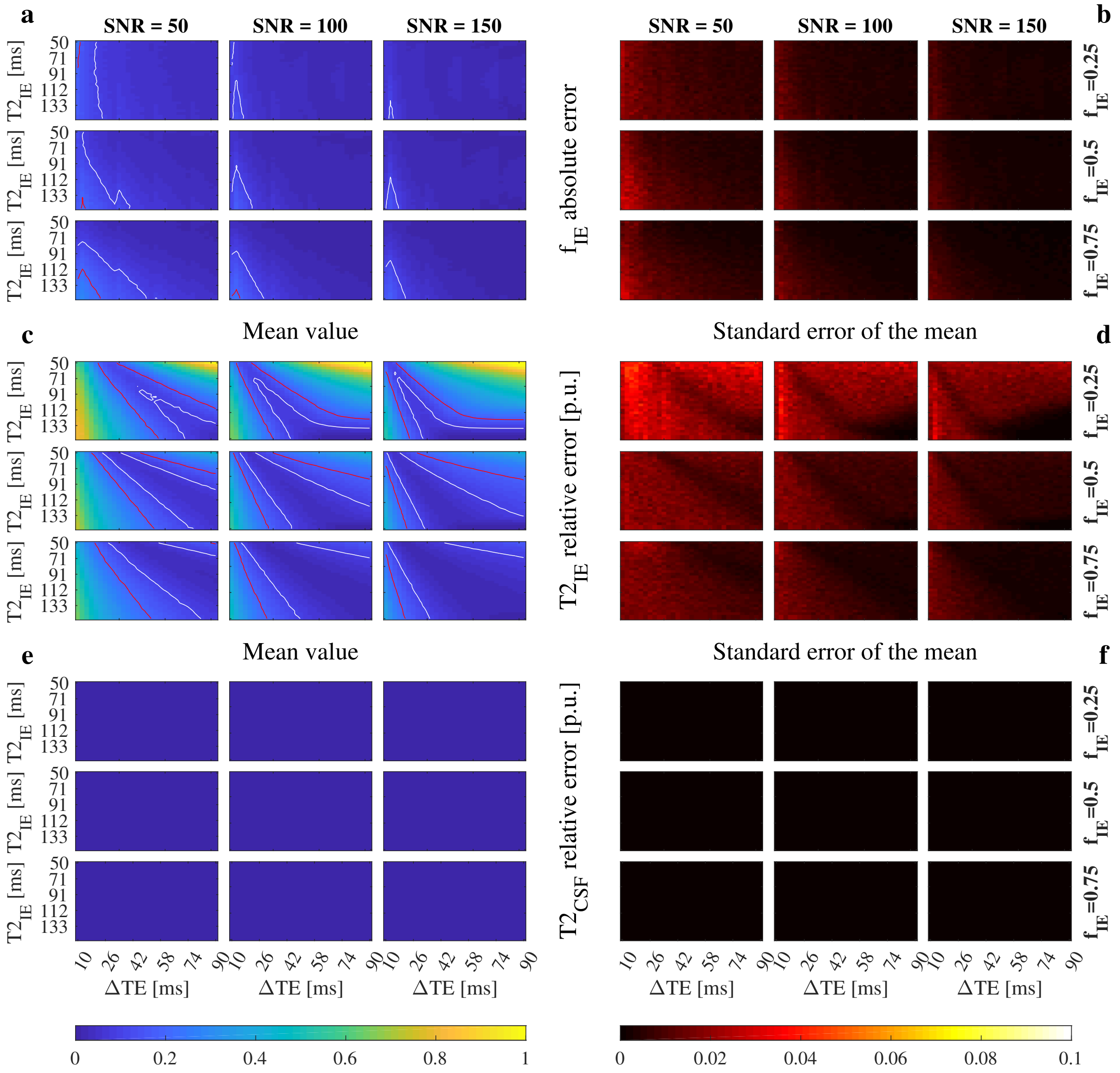

Figure S12: **Convergence for two compartments (IE and CSF) with fixed $T2_{CSF}$ and no S_{CSF} prior.**

The mean and the standard error of f_{IE} absolute error (a and b), and the mean and the standard error of $T2_{IE}$ (c and d), and $T2_{CSF}$ (e and f) relative error per unit (p.u.). Red and white lines mark the 0.2 and 0.1 contour respectively. One thousand simulations were run for each combination of SNR, f_{IE}, $T2_{IE}$, and ΔTE. $T2_{IE}$ was bound between 0–300 and $T2_{CSF}$ fixed to 2000 ms. No prior was imposed on S_{CSF}. We defined the convergence area as the one with error lower than 0.1 for f_{IE} and $T2_{IE}$. Fixing the value of $T2_{CSF}$ does not have any effect on the size of the convergence area, while bounding $T2_{IE}$ does it (see Figure S11).

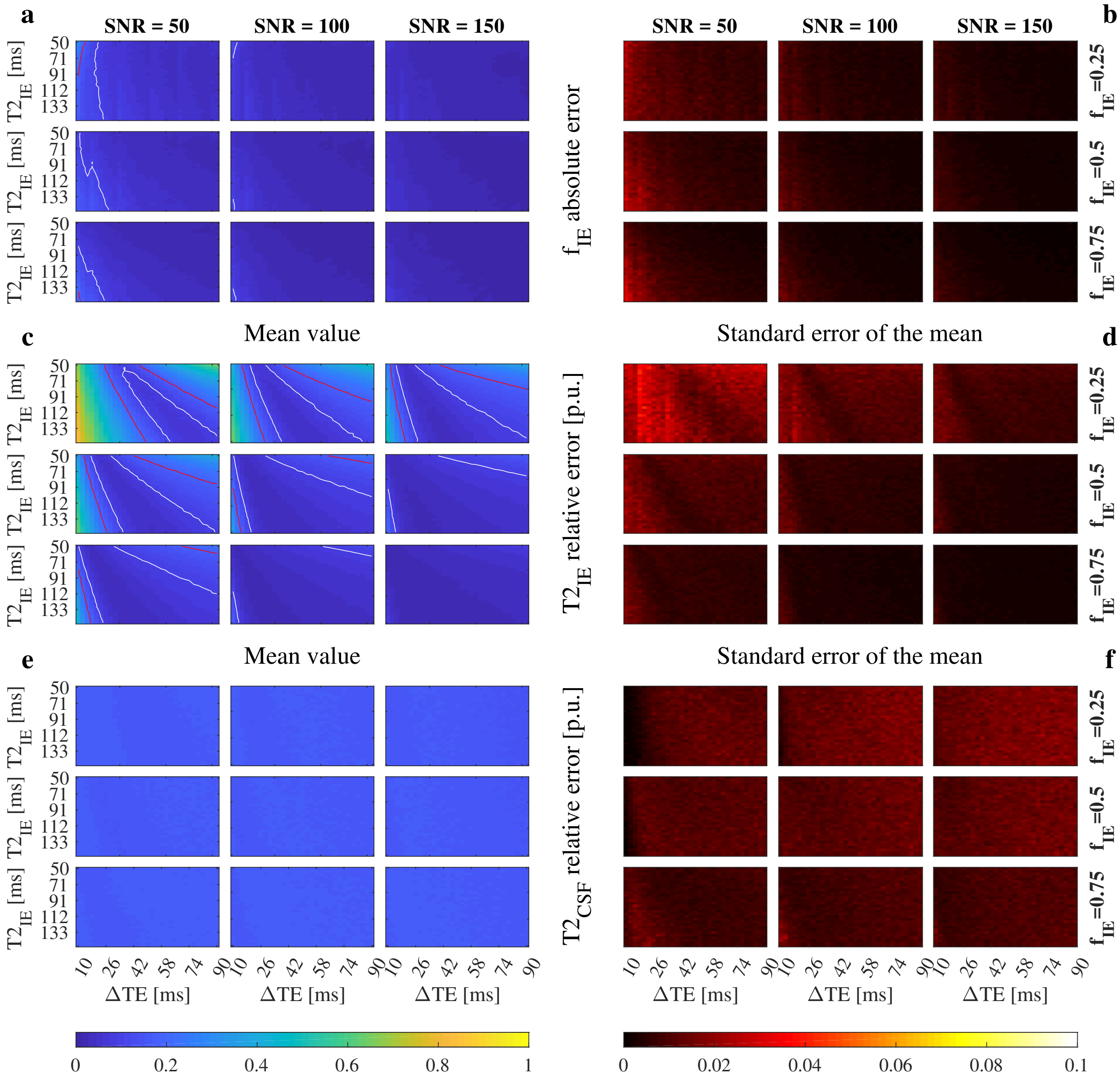

Figure S13: **Convergence for two compartments (IE and CSF) with non-overlapping T_2 constraints and S_{CSF} prior.**

The mean and standard error of f_{IE} absolute error (a and b), and mean and standard error of $T_{2_{IE}}$ (c and d), and $T_{2_{CSF}}$ (e and f) relative error per unit (p.u.). Red and white lines mark the 0.2 and 0.1 contour respectively. One thousand simulations were run for each combination of SNR, f_{IE}, $T_{2_{IE}}$, and ΔTE. $T_{2_{IE}}$ and $T_{2_{CSF}}$ were bound between 0–300 ms and 300–3000 ms respectively. S_{CSF} was set to have isotropic diffusivity with value 3×10^{-3} mm^2/s. We defined the convergence area as the one with error lower than 0.1 for f_{IE} and $T_{2_{IE}}$. Incorporating prior knowledge on the behavior of the signal sources (as CSF) improves convergence and stability more than bounding T_2 (Compare with Figures S10 and S11)

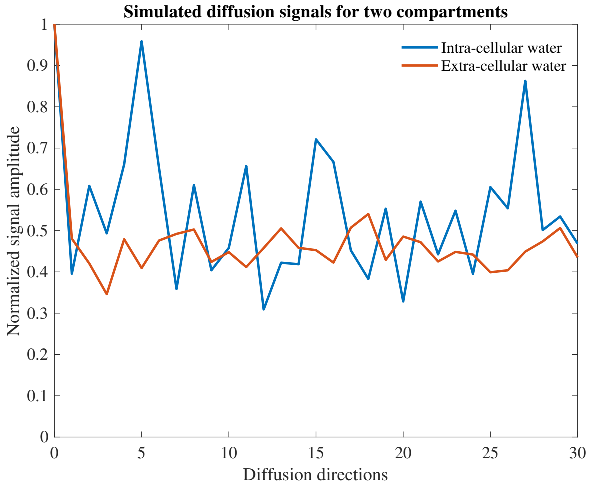

Figure S14: **Simulated diffusion signals for intra and extra-cellular water compartments.** Synthetically generated diffusion signals for 30 directions (b = 1000 s/mm^2) and one non-diffusion weighted measurement. We modeled diffusion as a Gaussian process with MD of intra-cellular (IC) and extra-cellular (EC) equal to 0.6×10^{-3} and 0.8×10^{-3} mm^2/s respectively (to keep the MD of parenchyma equals to 0.7×10^{-3} mm^2/s (28)) and standard deviations of 0.3×10^{-3} and 0.1×10^{-3} mm^2/s respectively to distinguish between a more (IC) and less (EC) hindered anisotropic diffusivity.

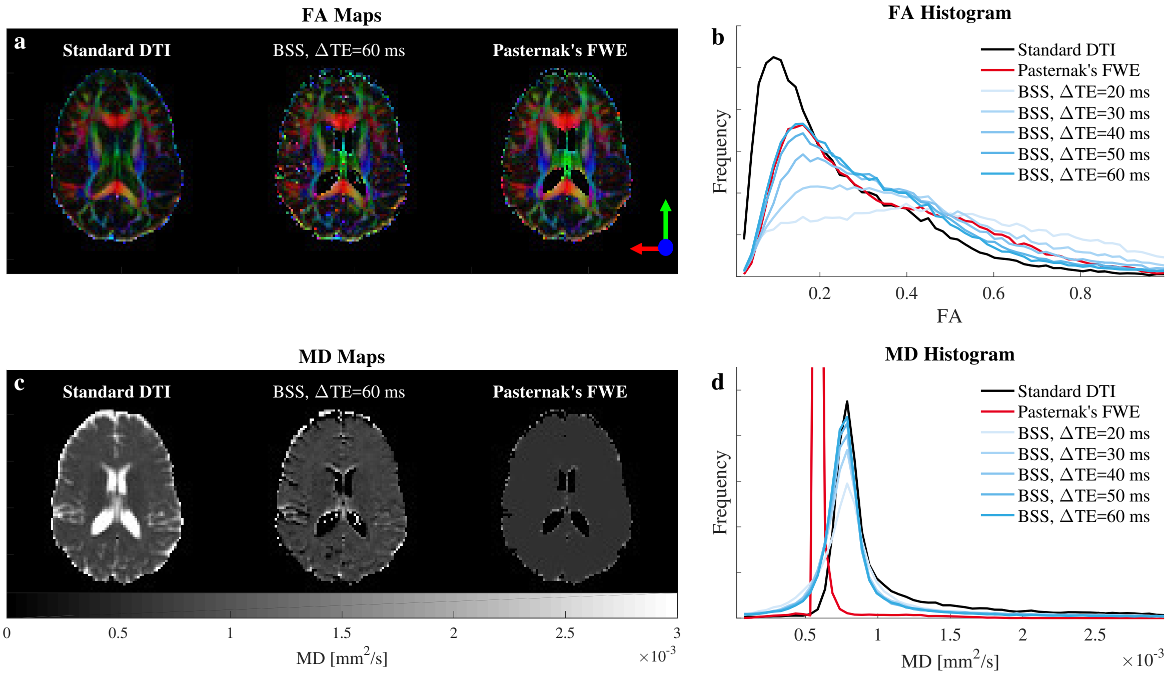

Figure S15: **FA and MD of the BSS-disentangled IE signal against the standard DTI and Pasternak's free-water elimination (FWE) for subject one.**
Comparisons of the FA (b) and MD (d) histograms calculated from the separated IE signals are plotted against the standard DTI fit and Pasternak's method for the short TE measured data. MD (c) and colored FA (a) maps are also included for comparison. We observed a CSF correction effect in the long ΔTE BSS for FA in agreement with Pasternak's FWE. However, both method disagree for MD, where Pasternak's introduces spatial over-regularization. See Figure 9 for subject two.

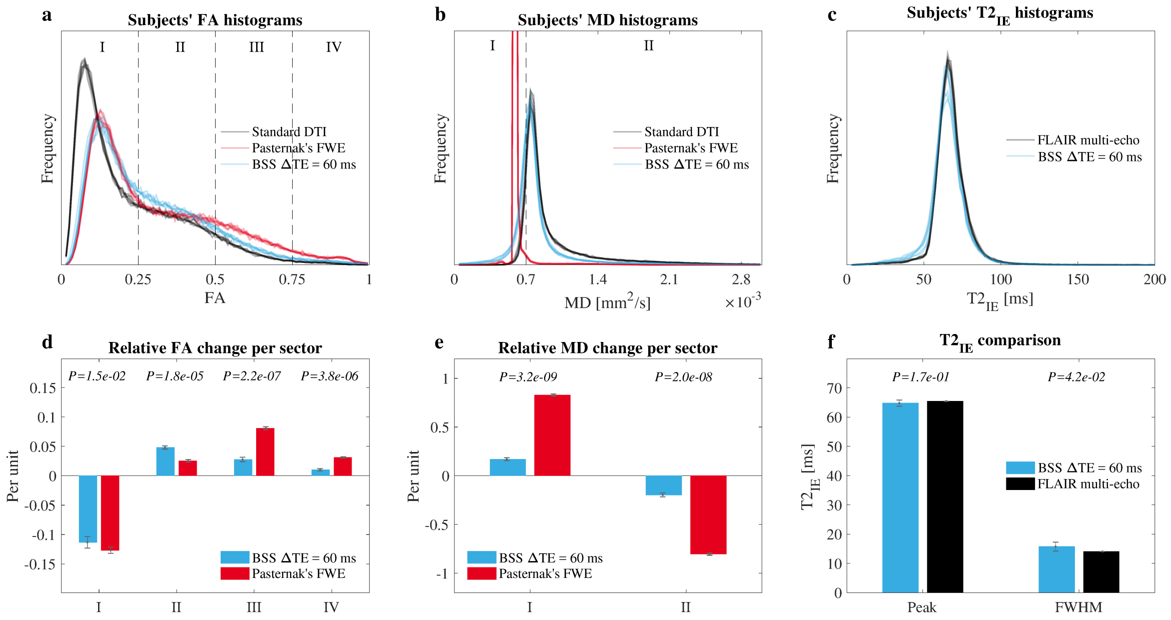

Figure S16: **Repeatability analysis showing intra-subject variability.**

A healthy volunteer was scanned six times. The FA (a) and MD (b) histograms for standard DTI, BSS and Pasternak's method are shown. These histograms were fragmented in sectors and the relative changes in number of voxels per sector and repetition for BSS and Pasternak's methods were computed. Statistical t-tests were run per sector to determine the level of significance of the differences between BSS and Pasternak's results (d and e). BSS and FLAIR $T_{2_{IE}}$ histograms (c) showed good agreement. Their peak and the full width half maximum (FWHM) were used for t-test comparison between BSS and FLAIR (f) highlighting the concordance.

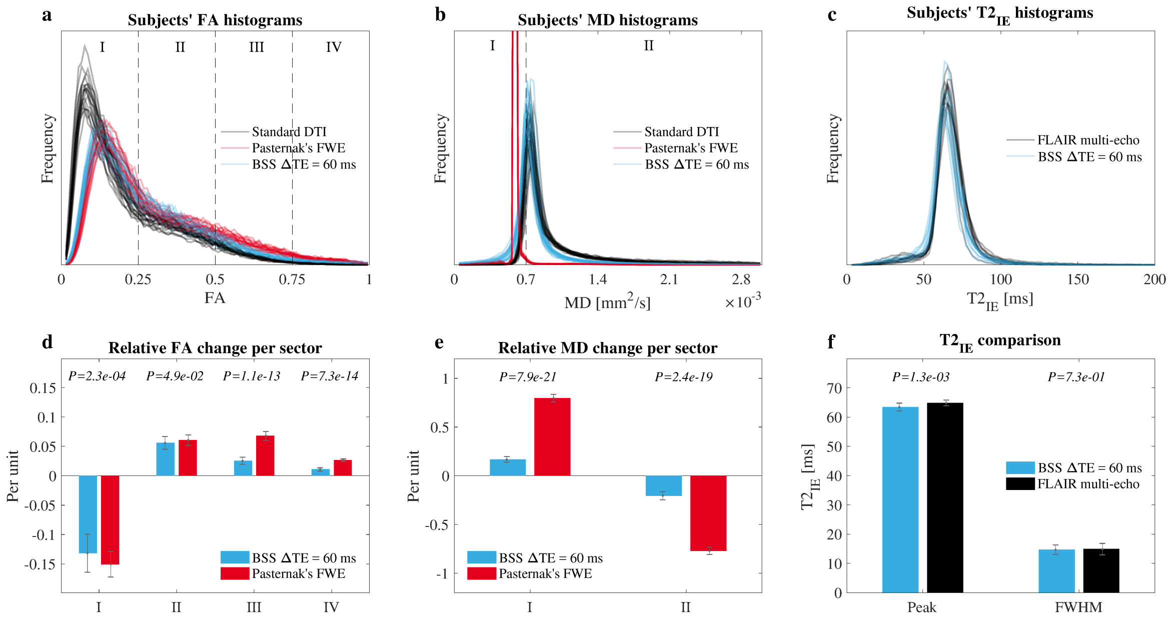

Figure S17: **Reproducibility analysis showing inter-subject variability.** Twenty healthy volunteers were scanned. The FA (a) and MD (b) histograms for standard DTI, BSS and Pasternak's method are shown. These histograms were fragmented in sectors and the relative changes in number of voxels per sector and repetition for BSS and Pasternak's methods were computed. Statistical t-tests were run per sector to determine the level of significance of the differences between BSS and Pasternak's results (d and e). Notice that the inter-subject variability is larger than intra-subject (Figure S16). BSS and FLAIR $T2_{IE}$ histograms (c) were depicted. Their peak and the full width half maximum (FWHM) were used for t-test comparison between BSS and FLAIR (f).
